# Identification of new members of the MAPK gene family in plants shows diverse conserved domains and novel activation loop variants

**DOI:** 10.1186/s12864-015-1244-7

**Published:** 2015-02-06

**Authors:** Tapan Kumar Mohanta, Pankaj Kumar Arora, Nibedita Mohanta, Pratap Parida, Hanhong Bae

**Affiliations:** School of Biotechnology, Yeungnam University, Daehak Gyeongsan, Gyeonsangbook 712749 Republic of Korea; Department of Biotechnology, North Orissa University, Sri Ramchandra Vihar, Takatpur, Baripada, Mayurbhanj, Orissa 757003 India; Center for Studies in Biotechnology, Dibrugarh University, Dibrugarh, Assam 786004 India

**Keywords:** Mitogen activated protein kinase (MAPK), Activation loop, Conserved motifs, Orthologs, Paralogs, Phylogeny, Evolution

## Abstract

**Background:**

Mitogen Activated Protein Kinase (MAPK) signaling is of critical importance in plants and other eukaryotic organisms. The MAPK cascade plays an indispensible role in the growth and development of plants, as well as in biotic and abiotic stress responses. The MAPKs are constitute the most downstream module of the three tier MAPK cascade and are phosphorylated by upstream MAP kinase kinases (MAPKK), which are in turn are phosphorylated by MAP kinase kinase kinase (MAPKKK). The MAPKs play pivotal roles in regulation of many cytoplasmic and nuclear substrates, thus regulating several biological processes.

**Results:**

A total of 589 MAPKs genes were identified from the genome wide analysis of 40 species. The sequence analysis has revealed the presence of several N- and C-terminal conserved domains. The MAPKs were previously believed to be characterized by the presence of TEY/TDY activation loop motifs. The present study showed that, in addition to presence of activation loop TEY/TDY motifs, MAPKs are also contain MEY, TEM, TQM, TRM, TVY, TSY, TEC and TQY activation loop motifs. Phylogenetic analysis of all predicted MAPKs were clustered into six different groups (group A, B, C, D, E and F), and all predicted MAPKs were assigned with specific names based on their orthology based evolutionary relationships with *Arabidopsis* or *Oryza* MAPKs.

**Conclusion:**

We conducted global analysis of the MAPK gene family of plants from lower eukaryotes to higher eukaryotes and analyzed their genomic and evolutionary aspects. Our study showed the presence of several new activation loop motifs and diverse conserved domains in MAPKs. Advance study of newly identified activation loop motifs can provide further information regarding the downstream signaling cascade activated in response to a wide array of stress conditions, as well as plant growth and development.

**Electronic supplementary material:**

The online version of this article (doi:10.1186/s12864-015-1244-7) contains supplementary material, which is available to authorized users.

## Background

During evolution, plants have developed complex arrays of defense mechanisms to mitigate the copious, often adverse and ever changing environmental conditions. Perception of variations in environmental as well as internal developmental cues, transduction and amplification of signals and activation of the response to stimuli is crucial for survival, optimal growth and development. Protein kinases are important signaling molecules that perceive various signals and transduce them for active responses. These compounds carryout diverse phosphorylation processes at the transcriptional, translational and post-translational level by catalyzing the addition of phosphate groups to serine and threonine/tyrosine residues in their target proteins in both prokaryotic and eukaryotic cells [1,2]. These modifications have led to changes in catalytic activity, affinity and interaction activity of target protein. However, the phosphorylation events in proteins are reversible due to protein phosphatase, enabling maintenance of the balance between kinase driven phosphorylation and phosphatase driven dephosphorylation events [3].

Plant genomes are rich in genes that encodes protein kinases and constitute the kinase super-family [4]. These super families are divided into different classes based on amino acid sequence similarity and functional characteristics. The mitogen activated protein kinase gene family, which is one such family, is known for evolutionary conservation across eukaryotic taxonomic groups and functioning within hierarchical cascades [1]. Phosphorylated proteins carry out a wide array of cellular responses, including changes in gene expression, innate immunity, developmental programmes and stress and hormonal responses [5-8].

Mitogen activated protein kinases consist of three kinase-modules composed of mitogen activated protein kinase kinase kinase (MAPKKKs), mitogen activated protein kinase kinase (MAPKKs) and mitogen activated protein kinase (MAPKs). In the general model, extracellular signals activate MAPKKKs, which phosphorylate downstream MAPKKs. The phosphorylated MAPKKs in turn phosphorylate MAPKs [9,10]. Protein phosphorylation events may occur throughout the protein kinase sequences, but usually occur on the activation loop [11]. The activation loop, which is present at the C-terminal end, resides within sub-domain VII and VIII of sub-domain eleven [4]. The activation loop contains conserved serine, threonine and/or tyrosine amino acid residues that may be reversibly phosphorylated [6] via *cis* auto-phosphorylation or *trans* phosphorylation by upstream kinases [12].

The initial descriptions of components of the MAPK cascade have been provided for the popular model plant, *Arabidopsis*. Advancements in sequencing technologies and bioinformatics tools have greatly increased the pace of genome sequencing projects, resulting in successful sequencing of several plant genomes. Post genome sequencing projects have enabled relatively easy identification of particular gene families based on conserved signature motifs and sequence similarity. Available genome sequences from several plants genomes have provided us with an opportunity to identify MAPK family members across photosynthetic eukaryotes (plants and algae) that will shed more light on MAPK evolution and signaling in plants and lower photosynthetic eukaryotes. In recent years, identification of MAPK gene family members in plants has been limited to a few species including *Arabidopsis thaliana* [13], *Oryza sativa* [14], maize [15], *Brassica napus* [16], apple [17], and *Brachypodium* [18]. Further, a study by Janitza et al. [19] and a review article by Doczi et al. [20] have provided a comprehensive overview of the evolutionary history of MAPKs in green plants by using a limited number of plants species.

However, there is currently limited information regarding the nomenclature, conserved structures, genomics and biochemistry of MAPKs in plants. In this communication, we identify the MAPK gene families of 40 different plant species and provide a unique nomenclature to all MAPKs. This nomenclature system can be further applied to newly identified MAPKs of other species. Furthermore, the genomics, biochemistry and conserved consensuses of plant MAPKs describe several novel aspects of plant MAPKs.

## Results

### Identification and nomenclature of MAPKs

We identified the MAPK gene family from 40 different plant species starting from the unicellular lower eukaryote *Chlamydomonas reinhardtii* to the multi-cellular angiosperm *Arabidopsis thaliana* and attempted to cover the maximum number of species across the plant lineage. We found that MAPKs members of a genome varied from species to species across the whole plant lineage. The 40 species collectively gave rise to 589 MAPK sequences. The tetraploid *Glycine max* contained the most MAPK genes in its genome (31), whereas the lower eukaryotic plant *Ostreocccus lucimarinus* contained three (Table 1). In addition, *Brassica compestris* (30), *Gossipium raimonddi* (28), *Malus domestica* (28), *Panicum virgatum* (27), *Linum usitatissimum* (24) and *Populus trichocarpa* (21) contained higher number of MAPKs (Table 1, Additional file 1). All the identified 589 MAPKs were provided with specific names according to the orthologous sequence similarity with *Arabidopsis thaliana* or *Oryza sativa*.

**Table 1:** **Table representing genome size of different plant species and number of MAPK genes present per genome (species)**

**Sl. No**	**Name of plant species**	**Abbreviation of MAPKs**	**Type of organism**	**Ploidy level**	**Genome size (Mbs)**	**Total No. of loci**	**Total No. of MAPK genes**
1	*Aquilegia coerulea*	AcMPK	Dicot	Diploid	302	24823	10
2	*Arabidopsis thaliana*	AtMPK	Dicot	Diploid	135	27416	20
3	*Brachipodium distachyon*	BdMPK	Monocot	Diploid	272	26552	16
4	*Brassica rapa*	BrMPK	Dicot	Diploid	283.8	26374	30
5	*Capsella rubella*	CrMPK	Dicot	Diploid	134.8	26521	18
6	*Carica papaya*	CpMPK	Dicot	Diploid	135	27332	9
7	*Chlamydomonas reinhardtii*	CreinMPK	Algae	Haploid	111+ 7.8	12264	6
8	*Citrus clememtina*	CcMPK	Dicot	Diploid	301.4	24533	12
9	*Citrus sinensis*	CsMPK	Dicot	Diploid	319	25376	12
10	*Coccomyxa subellipsoidea*	CsubMPK	Algae	Haploid	49	9629	4
11	*Cucumis sativus*	CsMPK	Dicot	Diploid	203	21494	14
12	*Eucalyptus grandis*	EgMPK	Dicot	Diploid	691	36376	13
13	*Fragaria vesca*	FvMPK	Dicot	Diploid	240	32831	11
14	*Glycine max*	GmMPK	Dicot	Tetraploid	975	54175	31
15	*Gossipium raimondi*	GrMPK	Dicot	Diploid	761.4	37505	28
16	*Linum usitatissimum*	LuMPK	Dicot	Diploid	318.3	26374	24
17	*Malus domestica*	MdMPK	Dicot	Diploid	881.3	26.374	28
18	*Manihot esculenta*	MeMPK	Dicot	Diploid	533 (760)	30666	17
19	*Medicago truncatula*	MtMPK	Dicot	Diploid	241 + 16.6	44135	17
20	*Micromonas pusila*	MpMPK	Algae	Haploid	22	10660	4
21	*Mimulus guttatus*	MgMPK	Dicot	Diploid	321.7	26718	6
22	*Oryza sativa*	OsMPK	Monocot	Diploid	372	39049	17
23	*Ostreococcus lucimarinus*	OlMPK	Algae	Haploid	13.2	7796	3
24	*Panicum virgatum*	PvMPK	Monocot	Tetraploid	1358	65878	27
25	*Phaseolus vulgaris*	PvMPK	Dicot	Diploid	521.1	27197	14
26	*Physcomitrella patens*	PpMPK	Bryophyte	Haploid	480	32272	8
27	*Picea abies*	PaMPK	Gymnosperm	Diploid	1960	28354	14
28	*Populus trichocarpa*	PtMPK	Dicot	Diploid	422.9	41335	21
29	*Prunus persica*	PperMPK	Dicot	Diploid	227.3 + 224.6	27864	12
30	*Ricinus communis*	RcMPK	Dicot	Diploid	400	31221	12
31	*Selaginella moellendorffii*	SmMPK	Pteridophyte	Haploid	212.5	22273	6
32	*Setaria italica*	SiMPK	Monocot	Diploid	405.7	35471	16
33	*Solanum lycopersicum*	SlMPK	Dicot	Diploid	900	34727	17
34	*Solanum tuberosum*	StMPK	Dicot	Diploid	800	35119	12
35	*Sorghum bicolor*	SbMPK	Monocot	Diploid	697.5	34496	16
36	*Thellungiella halophila*	ThMPK	Dicot	Diploid	238.5	26351	16
37	*Theobroma cacao*	TcMPK	Dicot	Diploid	330.8	29452	12
38	*Vitis venifera*	VvMPK	Dicot	Diploid	487	26346	12
39	*Volvox carteri*	VcMPK	Algae	Haploid	125.4	14971	5
40	*Zea mays*	ZmMPK	Monocot	Diploid	2500	??????	19

From this table it is evident that, the number of genes in a specified gene family don’t directly proportional to its genome size.

### Genomics of MAPKs

Among 589 MAPKs identified from 40 different plant species, *Fragaria vesca* FvMPK20 contains the largest MAPK gene, with 2574 nucleotides long open reading frame (ORF), while *Panicum virgatum* PvMPK1-4 has the smallest gene, with 544 nucleotides long ORF (Additional file 1). Transcript organization showed that MAPKs have different arrays of intron organization in their genes. The numbers of MAPKs containing different arrays of introns were as follows: intronless (7), single intron (41), two introns (39), three introns (18), four introns (15), five introns (161), six introns (32), seven introns (20), eight introns (39), nine introns (126), ten introns (72) and eleven introns (18)(Additional file 1). The terrestrial plant *Selaginella moellendorffii* SmMPK10 contained maximum of 14 introns in its gene. Some intronless MAPKs present in higher eukaryotic plants include PvMPK7-2, PaMPK2, PaMPK3, PaMPK7-1, and PaMPK20, while lower eukaryotic algae contain OlMPK7 and OlMPK9 (Additional file 1).

The molecular weights of MAPK proteins were vary from 22.381 (VvMPK1) to 98.915 (MdMPK20-2) kDa and the isoelectric points vary from 5.00 (MdMPK20-1) to 9.52 (CsubMPK15) (Additional file 2). The isoelectric point (pI) of group A and group B MAPKs were ranges from acidic to slightly acidic, while those of group C and group D were reside within the basic pI ranges. The average amino acid composition of MAPK protein showed that, abundance of leucine (9.63) amino acid was maximum and tryptophan (0.70) amino acid was minimum (Additional file 3). The average abundance of the most important amino acids threonine, glutamic acid, and tyrosine (T-E-Y) were 4.65, 6.73 and 3.91, respectively, whereas the average abundance of aspartic acid was 6.01. The abundance of the hydrophobic amino acids alanine (6.91), isoluecine (6.24), leucine (9.63), phenylalanine (4.45), proline (6.25) and valine (5.70) in MAPKs were relatively higher than that of other amino acids (Additional file 3).

### Conserved motifs and domains

#### N-terminal conserved sequences

**Figure 1:**
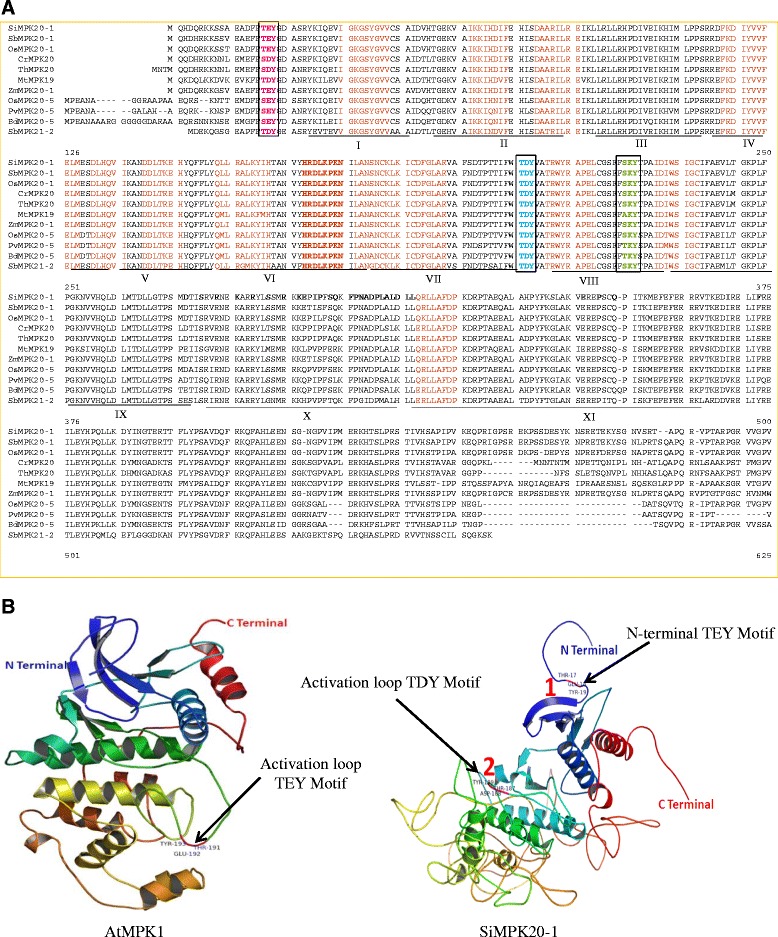
**Multiple sequence alignment of plant MAPKs. A.** The figure shows presence of N-terminal TEY, TDY, SEY and SDY motifs (in red). The SEY and SDY motifs are aligned with TEY and TDY motifs and very specific to group D MAP kinase genes. Activation loop TDY motifs are marked in blue and indicated inside the box. Conserved signature consensus sequences present within MAP kinases domain of MAPK proteins are marked in maroon. C-terminal conserved motifs are marked in green and presented inside box. All eleven sub-domains are also indicated in the figure. **B.** Molecular structure of AtMPK1 (group C) and SiMPK20-1 (group D). In AtMPK1, the arrow mark shows presence of TEY motif in the activation loop region. Similarly, in the group D MAP kinase (SiMPK20-1), the conserved N-terminal TEY motif and activation loop TDY motifs are indicated by different arrows.

**Figure 2:**
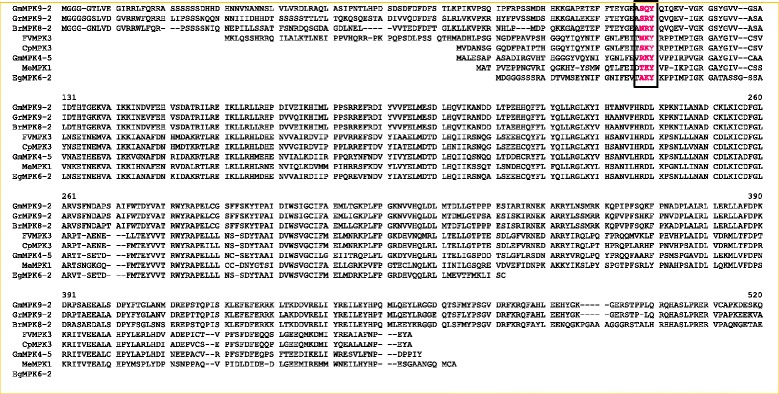
**The N-terminal conserved motifs of plant MAPKs.** These motifs are immediately followed by presence of N-terminal SEY, SDY, TEY, TDY motifs of group D MAPKs. This picture is a pictorial representation of different motifs taken from different groups of MAPKs. For details, please see Table 2 and Additional file 4.

The MAPKs are characterized by the presence of a conserved T-E-Y/T-D-Y motif in the activation loop region. Despite having the activation loop T-E-Y/T-D-Y motif in MAPKs, in this study, we found that several MAPKs shared conserved N-terminal T-E-Y, T-D-Y, S-D-Y and S-E-Y motifs (Figure 1A, 1B, Table 2, Additional file 4). These N-terminal conserved motifs are only shared by group D MAPKs. In total, 182 genes shared the N-terminal conserved motifs. Among them, 11 genes shared the S-D-Y motif, 27 shared the S-E-Y motif, six shared the T-D-Y motif and the remaining138 genes shared the T-E-Y motif (Additional file 4). *Chlamydomonas* and *Volvox* share a common A-V-H motif instead of the S-E-Y/S-D-Y/T-E-Y and T-D-Y motif (Additional file 4). Several other group specific conserved motifs are also present in the N-terminal region of MAPKs. They includes A-K-Y, N-K-Y (group A), S-K-Y, R-K-Y (group B), T-K-Y (group C) and S-Q-Y, N-R-Y, S-R-Y (group D) (Figure 2, Table 2). These motifs are present immediately after the N-terminal T-E-Y, T-D-Y, S-D-Y and S-E-Y motifs. The MAPK sequences sharing different numbers of motifs are A-K-Y (70), N-K-Y (13), S-K-Y (74), R-K-Y (42), T-K-Y (81), S-Q-Y (36), N-R-Y (98), and S-R-Y (91) (Additional file 4). In addition to the presence of conserved motifs, the N-terminal region of MAPKs also contained conserved amino acid consensus sequences including I-G*-*x*-*G-x-Y-G*-*x*-*V, I-K-K-I-x_3_-F, D-A-x*-*R-x-L-R-E*,* F-x*-*D-I-Y-x_3_-E-L-M, D-L-x_2_-V-I*,* D-x*-*L-x_2_-E-H*,* Q*-*x-L-R-x*-*L-K-Y-x-H*,* H*-*R-D-L-K-P-x*-*N*,* and L-x-L-x-N-C-x*-*L-K-I-x-D-F-G-L-A-R (Figure 1A, Table 3).

**Table 2:** **Different conserved motifs present in N-terminal, activation loop region and C-terminal end of plant MAPKs**

**Conserved signature motifs of MAPKs**			
N-Terminal conserved motifs	Some other conserved Motif Present in N-terminal End	Activation loop motifs	C-Terminal motifs
Group D	Group A: A-K-Y, N-K-Y	Group A: T-E-Y, T-Q-Y	Group A & B: S-D-Y, S-E-Y, T-D-Y
S-D-Y, S-E-Y	Group B: S-K-Y, R-K-Y	Group B: T-E-Y, M-E-Y, T-E-C, T-V-Y	Group C: D-N-Y, S-Q-Y
T-D-Y, T-E-Y	Group C: T-K-Y	Group C: T-E-Y	Group D: S-K-Y, T-K-Y, S-R-Y, S-N-Y
	Group D: S-Q-Y, N-R-Y, S-R-Y	Group D: T-D-Y	
		Group E: T-S-Y, T-E-M, T-Q-M, T-R-M

**Table 3:** **Table presenting different conserved consensus sequences present in plant MAPKs**

**Common conserved signature sequences of MAPKs**	
**N-Terminal conserved consensus sequences**	**C-Terminal conserved consensus sequences**
I-G-x-G-x-Y-G-x-V	T-R-W-Y-R-A-P-E-L
I-K-K-I-x_3_-F	I-D-x-W-S-I/V-G-C
D-A-x-R-x-L-R-E	Q-x-L-L-x-F-D-P
F-x-D-I-Y-x_3_-E*-*L-M	
D-L-x_2_-V-I	
D-x-L-x_2_-E-H	
Q*-*x-L-R-x-L-K-Y-x-H	
H*-*R-D-L*-*K-P-x-N	
L-x-N-x-N-C-x-L-K-I-x-D-F-G-L-A-R	

#### Conserved sequences in activation loop region

**Figure 3:**
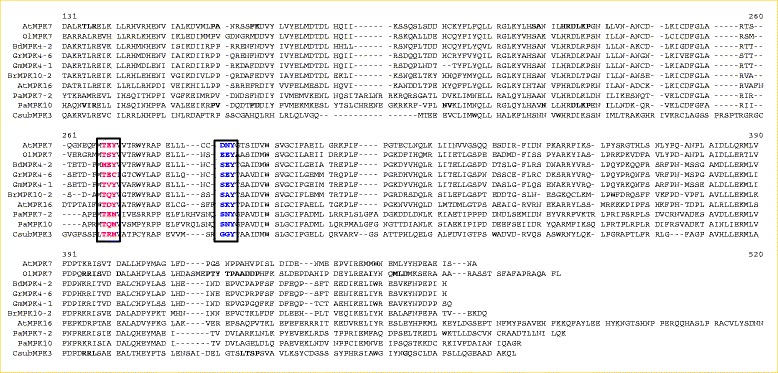
**Multiple sequence alignment of novel activation loop motifs of plant MAPKs.** Beside presence of TEY and TDY motifs in activation loop region, plant MAPKs show presence of other 8 different kinds of motifs (TSY, MEY, TEC, TVY, TQY, TEM, TQM and TRM) in the activation loop region as mentioned in figure (in red). These new motifs are aligned with conventional TEY and TDY motifs of AtMPK7 and AtMPK6 in activation loop region. This figure prepared to show the alignment of 8 newly identified motifs with TEY and TDY activation loop motif of MAP kinase. *Arabidopsis thaliana* AtMK7 and AtMPK16 were taken as representative of TEY and TDY motif respectively. Activation loop motifs are closely followed by presence of some group specific conserved motifs like DNY, EEY, SEY, SAY, SKY, SNY, GGY etc. These group specific motifs are indicated in blue color and present inside box.

**Figure 4:**
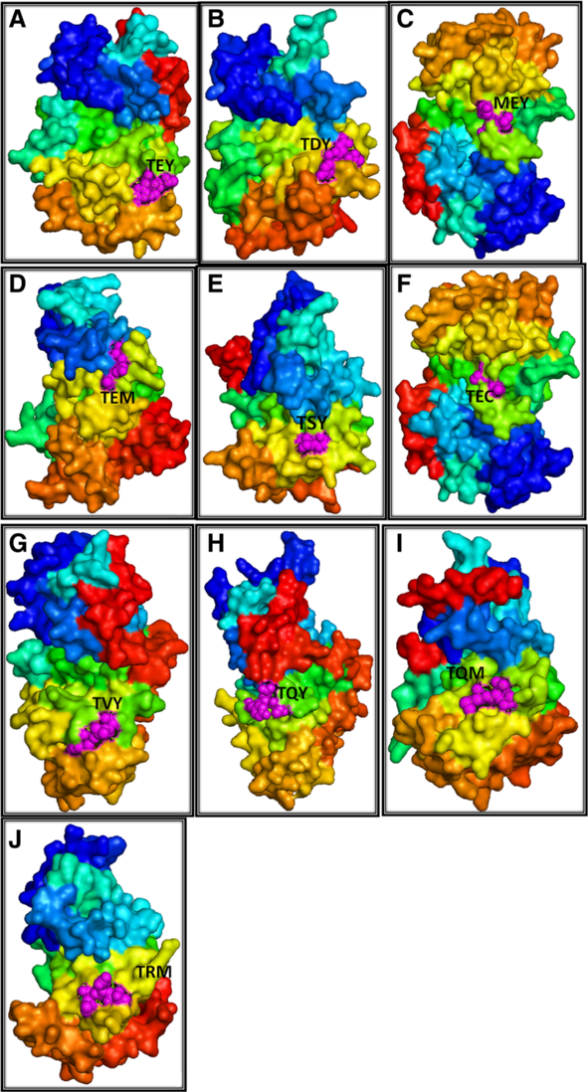
**Molecular structure of plant MAPKs showing their activation loop motif.** The structures were modeled by homology based modeling procedure using the Phyre2 server. The activation loop motifs were labeled using Pymol. Figures **A, B, C, D, E, F, G, H, I, J** represents T-E-Y (AtMPK1), T-D-Y (AtMPK20), M-E-Y (OsMPK16-2), T-E-M (PaMPK7-2), T-S-Y (OlMPK7), T-E-C (GrMPK4-6), T-V-Y (GmMPK4-1), T-Q-Y (BrMPK10-2), T-Q-M (PaMPK10) and T-R-M (CsubMPK3) motif respectively.

As discussed earlier, MAPKs contain the classic T-E-Y or T-D-Y motif in the activation loop region, and we found that majority of MAPK members contain classic T-E-Y/ T-D-Y motif in the activation loop region. Sequence alignment revealed that *M. domestica* MdMPK9 and *P. trichocarpa* PtMPK17-1contain an additional sequence of **T**AYKQYFLWTKLLTFMK**DY** and **T**VCVFLKPGFTFQCLI**DY** between the conserved T-D-Y motif (Additional file 4). In this study, we found eight novel activation loop motifs of MAPKs and reported here for first time. The newly identified activation loop motifs are, T-Q-Y (group A), M-E-Y, T-E-C, T-V-Y, (group B), T-E-M (group D), T-S-Y, T-Q-M, and T-R-M (group E) (Figures 3 and 4, Table 2). In total, eight MAPK genes share the M-E-Y motif in the classic T-E-Y and T-D-Y region (Additional file 4). The MAPK genes sharing the M-E-Y motif are *S. lycopersicum* SlMPK4-1, *S. tubersum* StMPK4-1, *B. distachyon* BdMPK4-2, *P. vulgaris* FvMPK4-2, *S. italica* SiMPK4-2, *Z. mays* ZmMPK4-2, *S. bicolor* SbMPK4-2 and *O. sativa* OsMPK16-2. These M-E-Y motifs fall under group B MAPKs. The new motif T-Q-Y (group A) is shared by BrMPK10-2, T-E-C (group B) by GrMPK4-6, T-V-Y (group B) by GmMPK4-1, T-E-M (group E) by PaMPK5, PaMPK14 and PaMPK7-2, while T-S-Y is shared by OlMPK7 (group E), T-Q-M (group E) by PaMPK10 and T-R-M (group E) by CsubMPK3 (Figure 3, Table 2).

#### C-terminal conserved sequences

The classical T-E-Y/T-D-Y motif at the activation loop region is followed closely by the presence of different C-terminal S-D-Y/S-E-Y/T-D-Y/D-N-Y/S-Q-Y/S-R-Y/S-K-Y/S-N-Y motifs (Figure 3, Table 2). These S-D-Y/S-E-Y and T-D-Y motifs are shared by group A and group B MAPKs. Additionally, the D-N-Y, S-Q-Y, and T-K-Y motifs are shared by group C MAPKs and S-K-Y, T-K-Y, S-R-Y, and S-N-Y motifs are shared by group D MAPKs (Table 2). The MAPK sequences sharing different numbers of C-terminal motifs include S-D-Y (57), S-E-Y (123), T-D-Y (8), D-N-Y (78), S-Q-Y (7), S-K-Y (217), S-R-Y (7), and S-N-Y (11). There are also conserved T-R-W-Y-R-A-P-E-L,I-D-x-W-S-V-G-C and Q-x*-*L-L-x*-*F-D-P consensus sequences present in the immediately post activation loop region of MAPKs (Figure 1A, Table 3).

#### Common docking domains

During mitogen activated protein kinase (MAPK) signaling, the ability of MAP2Ks to recognize their cognate MAPKs are facilitated by presence of short docking motif (D-site) that binds to its target complementary region on the MAPK. Similarly MAPKs are also contain short docking site that recognizes many downstream target proteins by utilizing the same strategy. From the studied MAPKs, we did not find presence of any unique and specific conserved docking domains for all groups of the MAPKs. Instead, conservation of the docking domains consensus is somewhat group specific (Table 4). The conserved docking domains of different MAPKs are K-M-L-T-F-D-P-K/R-Q/K-R-I-T-V-E-D/E-A-L (group A), K-M-L-V/I-F-D-P-x-K-R-I-I-V-D-E-A-L (group B K-M-L-I-F-D-P-S/T-K-R-I-S-V-T-E-A-L (group C) and L-L-E-R/K-L-L-A-F-D-P-K-D-R-P-T-A-E-E-A-L (group D) (Table 4).

**Table 4:** **Table showing predicted group specific common docking (CD) sites of plant MAPKs**

**Predicted common docking (CD) sites of MAPKs**	
Group A	K-M-L-T-F-D-P-K/R-Q/K-R-I-T-V-E-D/E-A-L
Group B	K-M-L-V/I-F-D-P-x-K-R-I-I-V-D-E-A-L
Group C	K-M-L-I-F-D-P-S/T-K-R-I-S-V-T-E-A-L
Group D	L-L-E-R/K-L-L-A-F-D-P-K-D-R-P-T-A-E-E-A-L

### Phylogeny

**Figure 5:**
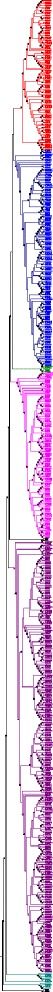
**Phylogenetic tree of MAPK gene family in plants.** Unrooted phylogenetic tree of MAPKs show presence of six different groups with well supported bootstrap values. We named them as group A (red), B (blue), C (pink), D (purple), E (teal) and F (green). The group E MAPKs are present at towards the end of the phylogenetic tree and group F present at the mid of the phylogenetic tree. Group E and F MAPKs are derived from lower plants of algae, pteridophytes and gymnosperm. Different MAPK genes falls in different groups are; group A (MPK3, MPK6, MPK10), group B (MPK4, MPK5, MPK11, MPK12, MPK13), group C (MPK1, MPK2, MPK7, MPK14), group D (MPK8, MPK9, MPK16, MPK17, MPK18, MPK19, MPK20 and MPK21), group E (CreinMPK7, CsubMPK3, PaMPK10, PaMPK7-2, PaMPK5, PaMPK14, CsubMPK7, MpMPK13, SmMPK10, VcMPK5) and group F (CreinMPK4-1, VcMPK4-1, OlMPK6, MpMPK4). The ERK1, ERK2, AtPIN1 (auxin efflux carrier) and AtCBL1 (calcineurin B like protein) were used as out group. Different statistical parameters used to constrcut the phylogenetic tree was: statistical method- maximum likelihood, test of phylogeny-boot strap method, no. of bootsrap replicate-2000, model/method-Jones-Taylor-Thornton (JTT) model, site coverage cutoff-95%, and branch swap filter-very strong. Phylogenetic tree was constructed using MEGA6 software.

An unrooted phylogenetic tree was constructed to infer group specific relationships of MAPKs. Upon phylogenetic analysis, all studied MAPK genes are fell into six different clusters, that are named according to the MAPK grouping of *A. thaliana*. In *A. thaliana*, MAPK genes are classified into four different groups (A, B, C, and D) based on their evolutionary relationship and presence of the T-D-Y and T-E-Y phosphorylation motif. In this study, MAPKs are categorized into six different groups namely group A (red), B (blue), C (pink), D (purple), E (teal) and F (green) (Figure 5, Additional file 5). Two new group of MAPK (group E and F) are generated during this analysis. The new group E and F are mainly shared by MAPKs of lower eukaryotic and gymnosperm plants such as CsubMPK7, MpMPK13, SmMPK10, CreinMPK7, VcMPK5, CsubMPK3, PaMPK10, PaMPK7-2, PaMPK5, PaMPK14, CreinMPK4-1, VcMPK4-1, OlMPK6, MpMPK4. The phylogenetic analysis revealed that 89, 128, 100, 258, 10 and 4 MAPKs fall into group A, B, C, D, E, and F respectively (Additional file 5, Table 5). The average overall phylogenetic mean distance of plant MAPK is 0.54 (standard error 0.029). During phylogenetic distance estimation, all the positions with less than 95% site coverage are eliminated. That is, fewer than 5% alignment gaps. The missing data and ambiguous bases are allowed at any position.

**Table 5:** **Table showing group specific distribution of different MAPKs in plants**

**Sl. No.**	**Name of plant species**	**Mitogen activated protein kinase**					
		**TEY**			**TDY**	**Other group**	**Total**
		**Group A**	**Group B**	**Group C**	**Group D**		
1	*Aquilegia coerulea*	3	2	3	2		10
2	*Arabidopsis thaliana*	3	5	4	8		20
3	*Brachipodium distachyon*	2	2	3	9		16
4	*Brassica rapa*	7	5	4	14		30
5	*Capsella rubella*	3	5	2	8		18
6	*Carica papaya*	2	2	1	4		9
7	*Chlamydomonas reinhardtii*	0	0	2	2	2	6
8	*Citrus clememtina*	2	3	2	5		12
9	*Citrus sinensis*	2	3	2	5		12
10	*Coccomyxa subellipsoidea*	0	0	1	1	2	4
11	*Cucumis sativus*	2	3	2	7		14
12	*Eucalyptus grandis*	3	3	1	6		13
13	*Fragaria vesca*	2	3	2	4		11
14	*Glycine max*	4	10	4	13		31
15	*Gossipium raimondi*	5	7	6	10		28
16	*Linum usitatissimum*	6	6	4	8		24
17	*Malus domestica*	5	8	5	10		28
18	*Manihot esculenta*	2	5	2	8		17
19	*Medicago truncatula*	2	4	5	6		17
20	*Micromonas pusila*	0	0	1	1	2	4
21	*Mimulus guttatus*	0	2	1	3		6
22	*Oryza sativa*	2	2	2	10	1	17
23	*Ostreococcus lucimarinus*	0	1	1	1		3
24	*Panicum virgatum*	4	4	4	15		27
25	*Phaseolus vulgaris*	2	4	2	6		14
26	*Physcomitrella patens*	0	4	2	2		8
27	*Picea abies*	1	2	5	2	4	14
28	*Populus trichocarpa*	4	3	4	10		21
29	*Prunus persica*	2	3	2	5		12
30	*Ricinus communis*	2	3	2	5		12
31	*Selaginella moellendorffii*	0	1	2	2	1	6
32	*Setaria italica*	2	2	2	10		16
33	*Solanum lycopersicum*	3	4	2	8		17
34	*Solanum tuberosum*	0	3	2	7		12
35	*Sorghum bicolor V 1.4*	2	2	2	10		16
36	*Thellungiella halophila*	3	4	2	7		16
37	*Theobroma cacao*	2	3	2	5		12
38	*Vitis venifera*	2	3	2	5		12
39	*Volvox carteri*	0	0	1	2	2	5
40	*Zea mays*	4	2	2	11		19

### Statistical analysis

Different statistical analysis was carried out to infer the statistical significance of the study. In Tajima’s relative rate test, different MAPK sequences were taken randomly from active data as different groups. Analysis was repeated for two times by taking random MAPK sequences into different taxonomic group A, B and C (these groups are statistical groups and should not be confused with MAPK groups). More specifically, group C was used as out group. When we took MgMPK4-1 (group A), GmMPK16-3 (group B) and AtPIN1 (group C), resulted *p-*value was 0.05935 and *X*^2^-test result was 3.56 (Table 6). When MgMPK4-1, GmMPK16-3 and AtCBL1 are taken as group A, B and C respectively, the *p-*value result was 0.00468 and *X*^2^-test result was 8.00. In both the cases statistical value was found to be significant (Table 6). The *P*-value less than 0.05 is often used to reject the null hypothesis of equal rates between lineages (p ≤ 0.01: very strong presumption against null hypothesis, 0.01 < p ≤ 0.05 strong presumption against null hypothesis, 0.05 < p ≤ 0.1 low presumption against null hypothesis, p > 0.1 no presumption against null hypothesis). The analysis involved 3 amino acid sequences. All positions containing gaps and missing data were eliminated. In Tajima’s test statistics (D) for neutrality, the D value was found to be 4.904140 (D = 4.904140) (Table 7). All the positions with less than 95% site coverage are eliminated during Tajima’s test for neutrality. There were a total of 322 positions in the final dataset.

**Table 6:** **Tajima’s relative rate test**

**Configuration**	**Count**	
	**MgMPK4-1, GmMPK16-3 AtPIN1**	**MgMPK4-1, GmMPK16-3 AtCBL1**
Identical sites in all three sequences	15	7
Divergent sites in all three sequences	64	44
Unique differences in sequence A	13	8
Unique differences in sequence B	5	0
Unique differences in sequence C	70	47
*P-*value	0.05935	0.00468
*X* ^2^ -test	3.56	8.00

Tajima’s relative rate test was carried out by randomly comparing three phylogenetically distant sequences in each case by distributing them into three distinct group. In column 2, group A (OsMPK6), B (PaMPK8) and C (SmMPK16-2); column 3, group A (AtMPK1), B (PaMPK8) and C (CreinMPK4-2); column 4, group A (SiMPK14), B (VcMPK9) and C (PaMPK3); column 5, group A (SbMPK14), B (PpMPK4-3), and C ( PaMPK3). Test was replicated for four times. In all the four cases, statistical result was found to be significant.

**Table 7:** **Tajima’s test for neutrality**

***m***	***S***	***p***_s_	***Θ***	***π***	***D***
594	320	0.993789	0.142719	0.377879	4.904140

The analysis involved 589 amino acid sequences. All positions with less than 95% site coverage were eliminated. That is, fewer than 5% alignment gaps, missing data, and ambiguous bases were allowed at any position. There were a total of 325 positions in the final dataset. Evolutionary analyses were conducted in MEGA6. *Abbreviations*: *m* = number of sequences, *n* = total number of sites, *S* = Number of segregating sites, *p*_s_ = *S*/*n*, *Θ* = *p*_s_/a_1_, *π* = nucleotide diversity, and *D* is the Tajima test statistic.

### Gene duplication

Chromosomes are evolved via fusion, fission, insertion, and duplication events, allowing evolution of chromosome size and number, and hence the genes. Gene duplication is the major force acting on the evolution of different species, and the gene families are groups of genes generated by duplication. The sizes of gene families reflect the number of duplicated genes, which are known as paralogs. Several plant MAPKs analyzed during this study were found to be duplicate genes resulting in several paralogous genes (Additional file 1). The plants with duplicated genomes give rise to more duplicated MAPKs relative to species with non duplicated genomes. Accordingly, species such as *G. max*, *G. raimondii,* and *M. domestica* contain more duplicated genes. Nevertheless, almost all species posses duplicated MAPKs in their genome. The gene duplication result of plant MAPKs those contained novel activation loop motif are reported in table (Table 8). All the MAPK resulted in z-score above four with 100% level of confidence.

**Table 8:** **Gene duplication analysis of some selective plant MAPKs that contain novel activation loop motif**

**Gene name**	**Representative motif**	**Z-score**	**Level of confidence (%)**
AtMPK1	T-E-Y	19.46	100
AtMPK20	T-D-Y	11.88	100
OsMPK16-2	M-E-Y	6.85	100
PaMPK7-2	T-E-M	7.92	100
OlMPK7	T-S-Y	8.48	100
GrMPK4-6	T-E-C	13.94	100
GmMPK	T-V-Y	13.67	100
BrMPK	T-Q-Y	7.91	100
PaMPK10	T-Q-M	13.41	100
CsubMPK3	T-R-M	7.75	100

Analysis was carried out by Pinda (pipeline for intraspecies duplication analysis). Analysis shows all the MAPKs that contain novel activation loop motif resulted in z-score above four. The Z-score above four is considered highly significant to be duplicated.

### MAPK groups

#### MAPKs in monocotyledonous plants

Among the 40 different plant species analyzed during this study, six were monocotyledonous plants (*B. distachyon, O. sativa, P. virgatum, S. italica, S. bicolour* and *Z. mays*) (Table 1). Our study revealed that *O. sativa* contains 17 MAPKs, not 15 as reported earlier. Among monocot species, *P. virgatum* contains the highest number of MAPKs in its genome (27).

#### MAPKs in dicotyledonous plants

The MAPK gene family of dicotyledonous plants has shown large variations among MAPK gene family members (Table 1). Among 40 different species investigated herein, 26 were dicot plants. This group contained as few as 6 MAPK gene in *M. guttatus* to as many as 31 in soybean (*G. max*). Investigation of MAPKs in *G. max* in another study using the HMM (hidden Markov model) approach also showed 35 MAPKs; however, four of them are indeed ‘MAPK-likes’ genes, making the actual MAPK number 31.

#### MAPKs in lower photosynthetic eukaryotes (Algae, Moss and Pteridophyte)

The lower photosynthetic groups includes four algae, one bryophyte, one pteridophyte and one gymnosperm species (Table 1). Multiple MAPKs were seen in several species of algae (Table 1). Our study also showed multiple MAPKs (TEY, 2/3/4 and TDY, 1/2) in unicellular and multi-cellular algae (Table 5). A genome survey of *S. moellendorffii*, a model lycophyte (non-seed vascular plant) and a primitive species revealed the presence of six MAPKs with single MAPKs in group A and B, whereas two MAPKs were present in group C and D. As a result, there are four MAPKs with the TEY motif and only two with the TDY motif. Among the mosses and algae, *P. patens* and *O. lucimarinus* lacks ‘group A’ type MAPKs, while *V. cartieri* lacks ‘group B’ and ‘group C’ type MAPKs and *C. reinhardtii* and *C. subellipsoidea* do not possess ‘group C’ MAPKs. Interestingly, none of the studied lower photosynthetic eukaryotes or land plants (both mono and dicot species) lacked ‘group D’ type MAPKs.

## Discussion

### Nomenclature and identification of MAPKs

It is very important to assign an appropriate and specific name to each member of the family to enable a thorough understanding of it. Therefore, we provided unique names to all 589 identified MAPKs across the plant lineage using the orthologous based nomenclature system proposed by Hamel et al. [21]. In the traditional naming system, names are assigned to gene(s) that are identified and cloned first, regardless of their similarities to other gene(s). For example, if someone cloned the ThMPK gene from *Thelluginella halophila* first, it named as ThMPK1, regardless of its orthologous similarity with other MPKs. Accordingly, if this ThMPK1 has orthologous similarity with AtMPK6, it should be named ThMPK6, but this does not happen. However, orthology lends the legitimacy to transfer of functional similarities from its ancestors [22-24]. As a result, orthology based nomenclature can provide succinct information regarding its orthologous counterpart gene. Practically, it is difficult to study every individual MAPK gene in all plant species to understand their specific roles in different aspects of plant biology. As orthology lends the legitimacy of common ancestry and evolutionary function, orthology based nomenclature will provide ideas regarding possible roles of specific genes in the plant species being investigated. This system of nomenclature can be further extended to newly identified gene families of other plant species.

To date, MAPK gene family members of only few plant species have been reported, including *Oryza sativa* [14], *Arabidopsis thaliana* [13], *Zea mays* [15], *Brachypodium distachyon* [18], Canola (*Brassica napus*) [16] and *Malus domestica* [17]. Although MAPKs from these plant species are previously identified, we have included them here to broaden the study. Inclusion of these species in our study led to identification of some new members of MAPKs. An earlier study by Hamel et al., revealed that the *O. sativa* genome contains 16 members of the MAPK gene family [21]. However, we recently identified 17 MAPK gene family members from *O. sativa*. Additionally, Zhang et al., reported 26 members of the MAPK gene family from *Malus domestica* [17], but we found that *M. domestica* contains 28 members.

### Genomics of MAPKs

All species are confined by specific numbers of fundamental traits known as chromosome [25,26]. The number of sets of chromosomes varies among genera and species, as well as within species. Some species are functionally haploid (e.g., *Chlamydomonas*, *Volvox, Coccomyxa*, *Ostreococcus*, *Physcomitrella*, *Selaginella*), or diploid (*Oryza sativa*, *Arabidopsis thaliana, Gossipium raimondii*, *Glycine max*, *Zea maize*) (Table 1). The genome size of a specific species is directly correlated with the ploidy level (haploid, diploid or polyploidy) of the organism [27,28]. The lower eukaryotic organisms such as *Chlamydomonas, Volvox, Coccomyxa, Ostreococcus, Physcomitrella,* and *Selaginella* are very simple life forms relative to higher eukaryotic angiosperms; therefore, they encode very few MAPK genes relative to higher plants. For example, the lower eukaryotic algae *O. lucimarinus* encodes only three MAPKs, while *C. subellipsoidea* encodes only four. *C. reinhardtii* and *S. moellendorffii* encode six MAPKs each, whereas *P. patens* encodes eight. The plants *G. max* has tetraploid genome; hence, its genome size is larger than those of *O. sativa* and *A. thaliana*. Owing to the ploidy nature of the genome, organisms encode more MAPKs [27,29,30]. *G. max* encodes maximum of 31 MAPKs, whereas *B. rapa* encodes for 30 MAPKs. Similarly, *M. domestica* and *G. raimondii* encodes for 28 MAPKs each. The presence of higher numbers of MAPK genes in these plants is attributed to their bigger genome size and ploidy level or whole genome duplication. The number of MAPK genes within each family varies from species to species depending upon the complexity and ploidy level of plants (Table 1).

During this study, we found that MAPK genes harbored several introns (Additional file 1). Additionally, different genes contained different numbers of introns ranging from zero (intronless) to fourteen. We did not find any group specific conserved intron organization for groups A, B, C, and E MAPK genes. However, group D MAPKs harbored seven to fourteen introns. It has been reported that intron organization is conserved at levels up to 10-20% between orthologs [31,32], and the presence of 126 MAPK genes containing nine introns in their gene constitutes 21.39% orthology. Accordingly, our findings are in agreement with earlier findings regarding the orthology based evolution of introns in plants. These findings confirm that group D MAPKs are evolutionarily more conserved than other groups of MAPKs.

### Conserved motifs and domains

Mitogen activated protein kinases are multigene families characterized by the presence of an activation loop T-E-Y/T-D-Y motif. [6,21] These conserved motifs are target phosphorylation sites of upstream MAPKK (mitogen activated protein kinase kinase).To date, there have been no reports regarding the presence of N-terminal conserved motifs in MAPKs. We found that group D MAPKs contains conserved N-terminal T-E-Y/T-D-Y/S-D-Y and S-E-Y motifs (Figure 1A, 1B, Table 2). *Chlamydomonas* and *Volvox* share a common A-V-H motif instead of the S-E-Y/S-D-Y/T-E-Y and T-D-Y motif. Protein phosphorylation can occur on multiple distinct sites throughput the given protein [33,34]. Hence the presences of N-terminal conserved motifs are may be target phosphorylation sites of some other unknown kinases. In addition to the presence of these conserved motifs, the N-terminal region of MAPKs also contains several other conserved consensus amino acids, I-G*-*x*-*G-x-Y-G*-*x*-*V, I-K-K-I-x_3_-F, D-A-x*-*R-x-L-R-E*,* F-x*-*D-I-Y-x_3_-E-L-M, D-L-x_2_-V-I*,* D-x*-*L-x_2_-E-H*,* Q*-*x-L-R-x*-*L-K-Y-x-H*,* H*-*R-D-L-K-P-x*-*N*,* and L-x-L-x-N-C-x*-*L-K-I-x-D-F-G-L-A-R (Figure 1A, Table 3). These conserved consensus amino acid sequences may be considered as signature consensus of MAPKs. The presence of conserved amino acids consensuses in MAPKs reflects their common evolutionary ancestry. Earlier, Hanks (2003) reported that the protein kinase super-family contains the G-x-G-x-x-G conserved consensus sequence [4], which is similar to the results of our study, in which I-G*-*x*-*G-x-Y-G*-*x*-*V was conserved instead of G-x-G-x-x-G across all MAPKs (Figure 1A, Table 3). In addition to the T-E-Y/T-D-Y activation loop motif of MAPK, several new and novel activation loop motifs were identified those includes M-E-Y (group B), T-E-M (group D), T-S-Y (group D), T-E-C (group B), T-V-Y (group B), T-Q-Y (group A), T-Q-M (group D) and T-R-M (group D) (Table 2). None of the identified motifs belonged to group C MAPK. These new motifs are assumed to be undergoing recent evolution to expand the diversity of MAPKs; however, the absence of a new kind of activation loop motif for group C MAPK indicates that this group is more conserved than other groups of MAPKs. The presence of diverse activation loop motifs are may be for target phosphorylation sites for diverse kinases that can facilitate phosphorylation events more easily to overcome selective pressure. These activation loop conserved motifs are closely followed by several other group specific conserved motifs, including S-D-Y, S-E-Y, T-D-Y (group A and B MAPKs), D-N-Y, S-Q-Y (group C MAPKs), S-K-Y, and T-K-Y (group D MAPKs) (Table 2). These motifs are may be putative phosphorylation sites of other kinases. The presence of the N-terminal conserved activation loop motif immediately followed by the presence of another conserved motif greatly reflects presence of wide arrays of phosphorylation sites of MAPKs, which may be target phosphorylation sites of different other kinases. The presence of N-terminal S-D-Y/S-E-Y/T-D-Y motifs along with C-terminal S-D-Y/S-E-Y/T-D-Y/S-Q-Y/D-N-Y/S-R-Y/S-K-Y/T-K-Y/A-K-Y/S-N-Y/G-R-Y motifs reflects challenges to defining MAPKs; however, these MAPKs can now be better defined as group specific. Although it has been proposed that the T-E-Y and T-D-Y motifs at the activation loop are important for activation of MAPKs, the presence of N-terminal and C-terminal S-D-Y/S-E-Y/T-D-Y as well as other motifs in close vicinity to the activation loop indicates that more in depth investigations are needed to confirm their functions. The presence of group specific conserved motifs and domains explains the orthology based evolution of MAPKs from the common ancestors.

In fungi, MAPKs regulates five different pathways namely CWI (cell wall integrity), HOG (high osmolarity glycerol), Kss, Fus, and Smk1 (sporulation and meiosis) [35]. The CWI pathway is carried out by fungal Mpk1 that contain T-E-Y motif in its activation loop domain, HOG pathway is carried out by Hog1 that contain T-G-Y motif, Kss pathway is responsible for filamentous growth in fungi and Kss1 contain T-E-Y motif, Fus pathway is responsible for mating and cell cycle arrest and contain T-E-Y motif in its activation loop domain. The fungal Smk1 pathway responsible for meiosis and sporulation contain T-N-Y motif in its activation loop domain. From these report, it is evident that MAPK that contain different activation loop motif controls different pathways. Presence of several new activation loop motifs in plants may be responsible for some novel pathways which are yet to be elucidated.

### Common docking site

It was previously assumed that substrate specificity of an enzyme is determined by stereo-chemical complementarities with its active site [36-39]; however, these preferences are not stringent enough. It has since been found that, in addition to substrate target site preferences, many protein kinases uses dedicated modular protein-protein interaction docking domains, interactions involving binding of the surface of the catalytic domain, but distinct from catalytic active sites [40-42]. These docking grooves are bind to the short peptide docking motifs that are separated from the substrate motif. Although these two supplemental recognition strategies are not mutually exclusive, development of these alternative modes of recognition provides a very simple method to meet increasing evolutionary requirements [41]. Protein kinases often recognize their substrates and regulators through docking interaction sites that occurs outside the active site [43,44], and these interactions can help us to understand the kinase networks. The MAPKs contains group specific conserved docking domains, K-M-L-T-F-D-P-K/R-Q/K-R-I-T-V-E-D/E-A-L (group A), K-M-L-V/I-F-D-P-x-K-R-I-I-V-D-E-A-L (group B), K-M-L-I-F-D-P-S/T-K-R-I-S-V-T-E-A-L (group C) and L-L-E-R/K-L-L-A-F-D-P-K-D-R-P-T-A-E-E-A-L (group D) (Table 4). The presence of group specific docking domains in MAPKs suggests that different MAPKs targets are group specific. Similarly, the presence of group specific conserved docking domains suggests, evolution of MAPKs are orthologous based and are group specific.

### Phylogeny of MAPKs

To understand the evolutionary expansion of MAPKs belonging to 40 different plant species, an unrooted tree was constructed from alignments of their full length protein sequences. The analysis revealed that all MAPKs fell into six different groups with well supported bootstrap values. We named them as group A (red), B (blue), C (pink), D (purple), E (teal) and F (green) (Figure 5, Additional file 5). The clustering of MAPKs into different groups reflects their orthology based origin from common ancestors. The cluster of group E MAPKs belonged to lower eukaryotic organisms and fell in the distal end (basal part) of the phylogenetic tree (Figure 5, Additional file 5). The finding of group E MAPKs from lower green algae and other organisms fell as an independent group suggests that these MAPKs were evolved independently and diverged during evolution to higher eukaryotes. Independent grouping of group E and F MAPKs may also indicate lower eukaryotic specific functions and their independent evolution [45-47]. Although biological functions of all MAPKs are not yet understood, MAPKs of the same subgroup are likely to be involved in similar physiological responses, and hence similar functions. The presence of lower eukaryotic specific group F MAPKs in the middle of the phylogenetic tree may also reflects their roles in evolution of MAPKs by subsequent divergence and duplication. During speciation events, these MAPKs became diversified and evolved as multigene families from common ancestors. It has been reported that multiple members of specific gene families of a particular organism are the natural products generated from the evolutionary history experienced by an organism [48-51]. Accordingly, the presence of several members in a gene family reflects the succession of genomic rearrangement, and its expansion is due to extensive duplication and diversification that occurs during the course of evolution [52,53]. Expansion of the gene family may be involved in different developmental processes of plants [54-58].

### Statistical analysis

In the random sampling of MAPK sequences in Tajima’s relative rate test, we found significant *p-*value and *X*^2^-test result in both the studied groups. This implies that our study is statistically significant (Table 6). In Tajima’s test for neutrality, the Tajima’s D test result is 4.904140 (D = 4.904140) (Table 7). In Tajima’s D test, when D = 0, the average heterozygosity became equal to number of segregating sites and can be interpreted as expected variation is similar to observed variation [59,60]. The evolving population can be due to mutation-drift equilibrium and no evidence of selection. When D < 0, the average heterozygosity is lower than number of segregating sites [59,60]. In this case, it can be interpreted as rare alleles are present at very low frequencies and recent selective sweep led to population expansion after recent bottleneck that contain the linkage to swept a gene. When D > 0, the average heterozygosity is more than that of segregating sites and can be considered as presence of multiple alleles; some at low and others at high frequencies [59,60]. This creates balancing in selection by sudden contraction in population. Tajima’s negative D value signifies excess low frequency of polymorphism relative to expectation. This indicates expansion in a population size by selective sweep or by purifying selection. Tajima’s positive D value signifies high frequencies of polymorphism indicating decrease in a population size by balancing selection. Tajima’s D value greater than +2 or less than −2 are considered as significant [59,60]. In our result we found D value of 4.904140 which is greater than 0 (D > 0) and +2 (Table 7). This signifies MAPKs are undergone high frequencies of polymorphism by decreasing population size due to balanced selection. So, the heterozygosity of plant MAPKs are more than that of number of segregating sites and presented as multiple alleles.

### Duplication of MAPKs

The size of the plant genome and number of chromosomes in each genome varies widely among species and shows diversity of 2350-folds ranges from 63 to 149,000 Mb that divided into n = 2 to n = ~ 600 chromosomes [25]. Chromosomes evolved by fusion, fission, insertion, and duplication events, allowing evolution of chromosome size and number and hence the genes [61,62]. Vascular plants evolved approximately 410 million years ago and diverged into several lineages, among which lycophytes, ferns and gymnosperm and seed plant survived [63]. The transitions from aquatic to land plants, gametophytic generation to sporophytic generation, non-vascular form to vascular form and non seed bearing to seed bearing life cycles requires evolution of new genes [63,64]. The co-linearity resulting from the common ancestry of angiosperms provides a powerful method to determine the orthology [65-67]. The MAPK genes from diverse species investigated in this study were found to be orthologous. Based on the results of this study, the orthologous MAPK genes have undergone duplications and given rise to several paralogous genes. The MAPKs those contained novel activation loop motif were found to be duplicated (Table 8). The most common MAPK (AtMPK1) that contain classical T-E-Y motif in its activation loop motif is highly duplicated with z-score 19.46 and 100% confidence level. The AtMPK20 that contained T-D-Y motif in its activation loop motif resulted in z-score of 11.88 with 100% confidence interval. All the newly identified MAPKs that contained novel activation loop motifs resulted in z-score more than four with 100% confidence level. Genes with z-score value four or more than four were considered as highly significant to be duplicated [68]. The phylogenetic study showed that MAPK genes of the grass family of monocotyledonous plants (*O. sativa, S. bicolor*, *B. distachyon, P. virgatum, S. italica* and *Z. mays*) are more conserved and clustered together, demonstrating that grass family MAPKs were evolved from a single lineage. It has been reported that, although genomes of the grass family differ greatly in terms of size, ploidy level and chromosome number, their genetic markers and genes are very well conserved between genomes [69,70]. It has also been reported that there are several duplicated MAPK genes in grass family plants due to their ploidy level and genome duplication [71,72].

Vascular plants appeared approximately 410 million years ago, then diverged into several lineages [73]. The first non-seed vascular plant, *Selaginella*, lacks evidence of whole genome duplication or polyploidy, which explain why *S. moellendorffii* contains only six MAPK genes. Although the genome sizes of *S. moellendorffii* and *A. thaliana* are very similar [63], *A. thalaiana* contains 16,574 (65%) duplicated genes [62]. The transition from a gametophyte to sporophyte dominant life cycle requires far fewer new genes than the transition from non-vascular non-flowering to vascular and flowering plants [63]. Earlier studies of *Chlamydomonas, Physcomitrella, Selaginella* and fifteen other angiosperm species by Banks et al., revealed that transition from single celled green algae to multi-cellular land plants requires 3006 new genes, but that transition from non-vascular to vascular plants is associated with a gain of only 516 genes [63]. They also reported that gene and genome duplication is pre-requisite for transition from simple leafless nonvascular sporophyte generation to dominant vascularized gametophyte generation and need almost three times as many new genes. The orthologous *Physcomitrella* and *Selaginella* shares around 84% to 89% of their genes in angiosperm plants, indicating their role as common ancestors [63]. These findings confirm that development from nonvascular to vascular life requires the stepwise addition of new genes directing their extra role in meristem development and hormonal signaling.

### MAPK groups

In this study, we identified MAPK gene family members from different plant groups including monocots, dicots and lower eukaryotic organisms. Overall, the plants included six monocots, 26 dicots, five algae, one bryophyte, one pteridophyte, and one gymnosperm. The amplification and diversification of the large MAPK family in the monocot plant *P. virgatum* could be largely attributed to its tetraploid nature (2n = 4× = 36) [17]. It will be interesting to determine whether the paralogs in a single group have attained diverse functions in *P. virgatum*. Among the studied monocots, *S. bicolour, S. italica* and *B. distachyon* were found to have 16 member MAPK families. Similar numbers of MAPKs in *Brachypodium* have been reported by Chen et al. [18].

Group A MAPKs of monocot plants are very stable orthologs of both AtMPK3 and AtMPK6, whereas none of any monocot genomes harbor orthologs of AtMPK10, suggesting that this MAPK may have been lost before the split of monocots. Group B MAPKs of monocot plants posses several paralogs of MPK4, suggesting that the duplication of MPK4 paralogs might have occurred before divergence of monocot plants. Group C MAPKs in monocots contain paralogs of MPK7 and MPK14, but lack any MAPK1 and MAPK2 gene (Table 5). However, a recent study reported the presence of ZmMPK1 and ZmMPK2 in maize, which are putative orthologs of AtMPK1 and AtMPK2, respectively [15]. Liu et al. showed that ZmMPK1 and ZmMPK2 are phylogenetically closer to AtMPK1 and AtMPK2 than AtMPK7 and AtMPK14, respectively, and thus suggested that naming these MAPKs as ZmMPK1 and ZmMPK2 would be more appropriate. This group C MAPK was previously named as ZmMPK7 [74]. However, evaluation of the evolutionary relationship of monocot MAPKs indicated that it would be more appropriate to name these MAPKs as ZmMPK7 and ZmMPK14. Group D MAPKs of monocot plants showed the presence of 10/11 MAPK genes and are relatively constant as compared to the 15 MAPKs in *P. virgatum* (Table 1). These findings indicate that the amplification of monocot group D MAPKs occurred before diversification of this species.

Group A MAPKs, which are relatively constant in monocots with two or four members and in dicot plants, showed zero to seven MAPK members. *M. guttatus*, which is phylogenetically closer to *Arabidopsis*, do not possess any group A MAPKs (Table 5). Another plant, potato (*S. tuberosum*), is particularly conspicuous in that it lacks the group A MAPK from its genome. *S. lycopersicum*, another member of solanaceae family, has three group A MAPKs in its genome. The absence of complete group A MAPKs in *M. guttatus* and *S. tuberosum* is intriguing, and more work in these plant systems is needed to reveal whether other groups of MAPKs have acquired many intrinsic functions of group A MAPKs in these plant species. Another member of group A, an ortholog of AtMPK10, was only observed in *B. rapa, T. halophila* and *C. rubella*, which all belong to the mustard family. These findings suggest that MPK10 orthologs are conserved only in the brassicaceae family of dicots, while it was lost from other families (except *Selaginella moelendorffii* and *Picea abies*) during evolution (Table 5).

The solanaceous plant *S. tuberosum* lacks group A MAPKs from their genome, suggesting that they have recently lost this group of MAPKs. The absence of group A MAPKs is particularly intriguing as it comprises orthologs of *Arabidopsis* MPK3 and MPK6. These two MAPKs show a high level of basal expression and even higher expression in response to biotic and abiotic stress, not only in *Arabidopsis*, but also in other plants. The roles of these two MAPKs in plant development have been well established in stomatal patterning [75], ovule development [76], seed formation and modulation of primary and lateral root development [77]. Definitive functions of group A MAPKs in growth and development and responses to biotic and abiotic stress have been shown in other plants as well [6,10,78-81]. Furthermore, double mutants of *mpk3 mpk6* show embryo lethality [10]. These studies in different plant systems suggest that MPK3 and MPK6 orthologs in different plant species are indispensable. It will be interesting to study these plants to address basic questions regarding MAPK signaling in response to different stress and developmental signals. Orthologs of AtMPK10 are confined to the dicot species *B. rapa, T. halophila* and *C. rubella*, which all belong to the mustard family. These findings suggest that MPK10 orthologs are only conserved in the brassicaceae family of dicots and have been lost from other families in the course of evolution (Table 5). The presence of four BrMPK10 paralogs might be due to duplication after *B. rapa* speciation since other mustard family members have single orthologs of MPK10. Similar to MPK3 and MPK6 orthologs, group A MAPKs are either present together or absent. The paralogs of MPK3 and MPK6 have been reported in several plant species. A recent report identified MAPKs in canola (*B. napus*) shows absence of MPK10 in its genome [16]. It is interesting to note that *B. napus* (AACC) is an allotetraploid between *B. rapa* (AA) and *B. oleracea* (CC). Evaluation of the expression using the ATH1 GeneChip (at *Arabidopsis thaliana* Kinase Database-AthKD) under control and different abiotic stress conditions revealed significant expression of AtMPK10 only in three stages/tissues from 208 tissues/stress conditions. These findings suggest a limited role of AtMPK10 in *Arabidopsis*. However, it will be interesting to investigate the functional relevance of paralogs of MPK10 in *B. rapa*. For group B MAPKs, with the exception of *A. thaliana*, no orthologs of AtMPK11 was observed in any of the studied dicot plant species, even in the mustard family (Table 5). In *Arabidopsis*, AtMPK11 showed significant expression in several tissues and under various stress conditions, suggesting it plays a specific role and will not likely to be lost or evolve into a pseudogene. Similarly, MAPK5, another group B MAPK is only restricted to brassicaceae family members with the exception of being present in *S. lycopersicum*. Eight of ten group B MAPKs in *G. max* are paralogs of MAPK4. Such a high number of paralogs in soybean suggests extensive events of duplication might have occurred in its genome or part of the genome. These findings are concurrent with recent reports that showed *G. max* underwent at least two putative genome wide and/or segmental duplications approximately 13 and 59 million year ago [82-86].

The group C MAPKs members in dicot plants were ranges from one to six (Table 5). The single members of the MAPK group were found in three dicot species, *E. grandis, M. guttatus* and *C. papaya*, in the form of either MPK7 or MPK1. The four orthologs of *Arabidopsis* (AtMPK1, AtMPK2, AtMPK7, and AtMPK14) are not restricted to a particular family or species of dicot plants. The group D MAPKs are unique owing to their ‘TDY’ motif in the activation loop and long C terminal common docking domain (Rodriguez et al., 2010 [10]). In dicot plants, the number of group D MAPKs varies from 2 in *A. coerulea* to 14 MAPKs in *B. rapa*. Among the studied dicot species, 21 showed less than ten group D MAPKs, suggesting that amplification of this group of MAPKs is more prominent in monocots.

We previously discussed loss of group A MAPK MPK10 from monocots and all dicots except members of the brassicaceae family. The lower eukaryotic plant *Selaginella* and gymnosperm plant *Picea abies* were found to contain MPK10, a group A MAPK. These findings suggested that MPK10 were existed in older species and are lost during divergence and speciation, after which it was only able to be transferred to members of the brassicaceae family. To identify the evolutionary path of MAPKs, Doczi et al., analyzed MAPK signaling components in evolutionarily representative species of a plant lineage from a free-living amoeba-flagellate protist, *Naegleria* (representative of an early diverging eukaryotic clade, Heterolobosea) to moss, algae and lycophytes [20]. Their study reported the presence of a single conventional MAPK in *Naegleria* with a ‘TEY’ motif, suggesting that the common ancestor of MAPKs were ERK-like, from which the distinct classes of MAPKs having TxY signature motifs were diverged [20]. Our study also showed multiple MAPKs (TEY, 2/3/4 and TDY, 1/2) in unicellular and multi-cellular algae (Table 5), suggesting that diversification events of MAPKs started in very early periods of the evolution of photosynthetic eukaryotes.

As the complexity of organisms increased, the MAPK family showed expansion via gene duplication [20]. The *P. patens*, a model moss species with a genome of 487 Mb [87], contains eight MAPKs, six of which were TEY type and two were TDY type (Table 5). The six TEY MAPKs were belonged to group B (four MAPKs) and group C (two MAPKs). Thus, on the evolutionary path of photosynthetic eukaryotes, lycophyetes, moss, multi-cellular and unicellular algae shows both TEY and TDY types of MAPKs. These findings suggest that the differences between these two types of MAPKs are ancient. Among mosses and algae, *P. patens* and *O. lucimarinus* lack ‘group A’ type MAPKs, while *V. cartieri* lacks ‘group B’ and ‘group C’ MAPKs, and *C. reinhardtii* and *C. subellipsoidea* do not possess ‘group C’ MAPKs. Interestingly, none of the studied species lacked ‘group D’ MAPKs. This observation suggests that the group D MAPKs or TDY MAPKs are indispensable in the lineage of green plants.

Overall, the MAPK gene family appears to be relatively constant in monocots (with the exception of *P. virgatum*), but not in dicots. Overall, the largest and smallest MAPK genes were observed in dicot plants.

## Conclusion

Genome wide analysis of MAPK gene family revealed presence of new activation loop motifs. The adopted MAPK nomenclature system can be extended to other plant species to maintain uniformity in the MAPK nomenclature system. Presence of novel activation loop motifs are new variants and could offer new type of gene regulation in plants.

## Methods

### Identification of MAPK gene family members

Mitogen activated protein kinase (MAPK) gene families from the model plant *Arabidopsis thaliana* were downloaded from The Arabidopsis Information Resources (TAIR: http://www.arabidopsis.org/) database [88]. The MAPK gene families from rice were downloaded from the TIGR rice Genome Annotation Resources (http://rice.plantbiology.msu.edu/) database [89]. The protein sequences of MAPKs from *Arabidopsis thaliana* and rice were used as search queries in the publicly available phytozome database (http://www.phytozome.net/) to identify MAPK genes in other plant species [90]. Overall, 40 species were included in this study and reported in Table 1. To identify MAPK gene families of unknown species, BLASTP searches was conducted using orthologous protein sequences *Arabidopsis thaliana* and *Oryza sativa* MAPK genes as the query search [91]. The genes identified through BLAST searches were used for further analysis. First, the top 100 genes were kept for systemic evaluation and indexing. The genes with serine/threonine protein kinase domains and the activation loop T-E-Y or T-D-Y motifs were considered as probable MAPK genes, which were subsequently confirmed by scanning in scan prosite and smart software for the presence of MAPK domain [92,93]. All datas were checked for redundancy and no any alternative splice variants were considered. Identified MAPK gene families from each species were again confirmed by running BLASTP searches against TAIR using the default parameters [92,93]. The genes were considered MAPK genes when BLASTP search matches with *Arabidopsis* MAPKs.

### Multiple sequence alignment and construction of quaternary structure

Multiple sequence alignment of MAPKs were carried out using the multiple sequence alignment tool Multalin (http://multalin.toulouse.inra.fr/multalin/) to identify the conserved domains. The following default parameters were used to run the multiple alignment programs: Multalin: fasta, protein weight matrix: Blosum62-12-12, gap penalty at opening: default, gap penalty at extension: default, gap penalties at extremities: none, one iteration only: no, high consensus value: 90% (default), low consensus value: 50% (default), maximum line length: 180, and graduation step: 10. Molecular structures of AtMPK1 (group C), SiMPK20-1 (group D) and other MAPKs those contain novel activation loop motif were constructed by Phyre [94] software and resulted PDB file was subjected PYMOL to indicate activation loop motif of MAPKs.

### Construction of phylogenetic tree

To construct the phylogenetic tree, all protein sequences were used to prepare a clustal file by running MAPKs protein sequences in clustal omega software using the following default parameters [95]: output format: clustal w/o numbers, dealign input sequences: no, MBED-like clustering guide tree: yes, MBED-like clustering iteration: yes, number of combined iterations: default (0), maximum guide tree iterations: default, maximum HMM (hidden Markov model) iterations: default, order: aligned. The generated clustal file of MAPKs was converted to MEGA file (.meg) format using the MEGA6 software and then employed to construct a phylogenetic tree [96]. The statistical parameters used to construct the phylogenetic tree were: analysis: phylogeny reconstruction; statistical method: maximum likelihood; test of phylogeny: bootstrap method; no. of bootstrap replications: 2000; substitution type-amino acids; model/method: Jones-Taylor-Thornton (JTT) model; rates among sites: uniform rates; gaps/missing data treatment: partial deletion; site coverage cutoff: 95%; ML heuristic method: nearest–neighbor-interchange (NII); initial tree for ML: make initial tree automatically (default-NJ/BioNJ); branch swap filter: very strong.

### Statistical analysis

Tajima’s relative rate test was carried out to understand the statistical significance between three different plant groups. The MEGA file used to construct phylogenetic tree was subjected to analyze Tajimas’s relative rate test. Random MAPK sequences were selected to analyze Tajima’s relative rate test in MEGA6 software. This random analysis was replicated for two times. Different statistical parameters used to analyze Tajima’s relative rate test were; scope: for three chosen sequence, substitution type: amino acid gaps/missing data treatment: complete deletion. On the other hand, Tajima’s test of neutrality was carried out to understand the evolution of randomly evolved MAPKs from none randomly evolved MAPKs. Different statistical parameters used to analyze Tajima’s test of neutrality were; scope: all selected taxas, substitution type: amino acid, gaps/missing data treatment: complete deletion.

The overall mean distances of all the MAPKs were calculated using MEGA6 software. The MEGA file used to construct phylogenetic tree was used to analyze the overall mean distance of plant MAPKs. Different statistical parameters used to calculate the overall mean distances were; analysis: distance estimation, scope: overall mean, variance estimation method: bootstrap method, no. of boot strap replication: 2000, substitution type: amino acid, model/method: p-distance, rate among site: uniform, pattern among lineage: same (homogenous), gaps/missing data treatment: partial deletion.

### Nomenclature of MAPKs

All predicted MAPKs were named based on the evolutionary relationship of MAPKs with *Arabidopsis thaliana* or *Oryza sativa* MAPKs as suggested by Hamel et al. [21]. During the nomenclature process, the first letter in upper case was used to identify the genus name, the second letter in lower case (in a few cases the first 2–3 letters) was used to identify species name, after which the MPK and number of corresponding orthologs of *Arabidopsis* or *Oryza sativa*. The monocot plant species were named according to the MAPK ortholog of *Oryza sativa*, while other species were named according to MAPK orthologs of *Arabidopsis thaliana*. If more than one ortholog was present in a particular species (or in the case of paralogs), the second number is followed by a hyphen to distinguish between paralogs.

## Additional files

Additional file 1:
**Additional data file showing the unified nomenclatured gene name, locus ID and detailed genomic information of plant MAPKs.**
Additional file 2:
**Additional data file showing the MAPK gene name, molecular weight (in kDa) and predicted isoelectric (pI) point.**
Additional file 3:
**Additional data file showing the average amino acid composition of plant MAPKs.**
Additional file 4:
**Additional data file showing multiple sequence alignment of all studied MAPK genes of 40 different plant species.**
Additional file 5:
**Additional data file showing the phylogenetic tree of plant MAPKs.** Red color indicates group A , blue color group B, pink color group C, purple color group D, teal color group E and green color represent group F MAPKs.

## Abbreviation

MAPK: Mitogen activated protein kinase
pI: Isoelectric point
kDa: Kilo dalton
