# Supplementary material for: Identification of new members of the MAPK gene family in plants shows diverse conserved domains and novel activation loop variants
Source: BMC Genomics. 2015 Feb 6;16(1):58. doi: 10.1186/s12864-015-1244-7 (PMC4363184; doi:10.1186/s12864-015-1244-7)
Supplement: Additional file 1: — Additional data file showing the unified nomenclatured gene name, locus ID and detailed genomic information of plant MAPKs. [file 12864_2015_1244_MOESM1_ESM.pdf]

Additional Data file 1:

Additional data file showing nomenclatured gene name, locus ID and detailed genomic information of plant MAPKs.

Gene Name	Locus ID	ORF	No. of a.a	No. of introns	5’-3’ Coordinate
		Aquilegia coerulea			
AcMPK1	Aquca_007_00283	1113	370	2	2556455 - 2559804
AcMPK2-1	Aquca_005_00492	1107	368	1	5729059 - 5731578
AcMPK2-2	Aquca_010_00559	1113	370	2	2556455-2559804
AcMPK3-1	Aquca_027_00128	1140	379	5	1306428 - 1310178
AcMPK3-2	Aquca_046_00022	1098	365	5	870772 - 875224
AcMPK4	Aquca_038_00076	1380	459	6	997376 - 1004837
AcMPK6	Aquca_030_00253	1212	403	6	1824600 - 1832012
AcMPK9	Aquca_041_00181	1869	622	10	1345264 - 1350567
AcMPK13	Aquca_027_00155	1101	366	5	1497577 - 1500634
AcMPK20	Aquca_022_00063	1857	618	9	877601 - 883215
		Arabidopsis thaliana			
AtMPK1	At1g10210	1113	370	2	3349219-3351180
AtMPK2	At1g59580	1131	376	2	21884043-21886051
AtMPK3	At3g45640	1113	370	5	16756770-16758698
AtMPK4	At4g01370	1131	376	5	567095-569088
AtMPK5	At4g11330	1131	376	5	6892055-6894149
AtMPK6	At2g43790	1188	395	5	18138307-18140988

AtMPK7	At2g18170	1107	368	2	7907804-7909923
AtMPK8	At1g18150	1770	589	11	6244384-6247769
AtMPK9	At3g18040	1533	510	10	6174719-6178465
AtMPK10	At3g59790	1182	393	5	22092448-22094240
AtMPK11	At1g01560	828	275	3	202136-204335
AtMPK12	At2g46070	1119	372	5	18945981-18947823
AtMPK13	At1g07880	765	254	4	2434030-2435763
AtMPK14	At4g36450	1086	361	1	17210245-17211413
AtMPK15	At1g73670	1731	576	10	277700050-27703440
AtMPK16	At5g19010	1704	567	9	6344789-6348212
AtMPK17	At2g01450	1461	486	9	199512-202306
AtMPK18	At1g53510	1848	615	9	19970664-19974393
AtMPK19	At3g14720	1797	598	9	4945601-4949042
AtMPK20	At2g42880	1821	606	9	17840388-17844380
Brachipodium distachyon					
BdMPK3	Bradi1g65810	1110	369	4	64739946 - 64742370
BdMPK4-1	Bradi3g32000	1128	375	6	34209148 - 34213422
BdMPK4-2	Bradi3g16560	1164	387	5	14742468 - 14745425
BdMPK6	Bradi1g49100	1176	391	5	47836748 - 47843578
BdMPK7-1	Bradi1g34030	1242	413	2	29632402 - 29634667
BdMPK7-2	Bradi4g24912	1110	369	1	30118480 - 30120192

BdMPK14	Bradi3g03780	1110	369	2	2521821 - 2525826
BdMPK16	Bradi2g36470	1635	544	9	36786589 - 36792652
BdMPK17	Bradi1g34700	1746	581	10	30307750 - 30312635
BdMPK20-1	Bradi2g44350	1848	615	9	44852084 - 44858179
BdMPK20-2	Bradi2g15317	1794	597	9	13689255 - 13693771
BdMPK20-3	Bradi1g41780	1266	421	8	38419711 - 38433437
BdMPK20-4	Bradi2g45870	1743	580	9	46302880 - 46308387
BdMPK20-5	Bradi2g16337	1746	581	9	14412074 - 14417497
BdMPK21-1	Bradi2g15620	1788	595	10	13875717 - 13880863
BdMPK21-2	Bradi2g45010	1734	577	13	45394037 - 45403577
Brassica rapa					
BrMPK1	Bra019955	1110	369	1	3519791 - 3521423
BrMPK2	Bra035437	1113	370	1	16278214 - 16279538
BrMPK3	Bra038281	1002	333	6	11394611 - 11396165
BrMPK4	Bra000955	1122	373	5	14117986 - 14119867
BrMPK5	Bra035233	1407	468	7	9400030 - 9402792
BrMPK6-1	Bra000326	1188	395	5	10551204 - 10553205
BrMPK6-2	Bra004784	1179	392	5	1814778 - 1816787
BrMPK7-1	Bra037234	1107	368	1	4863130 - 4864417
BrMPK7-2	Bra039629	1107	368	1	1590192 - 1591682
BrMPK8-1	Bra031017	1587	528	9	32925505 - 32928224

BrMPK8-2	Bra025929	1746	581	10	6952927 - 6956039
BrMPK9	Bra022276	1785	594	10	18688706 - 18691908
BrMPK10-1	Bra014527	1182	393	6	1220051 - 1222076
BrMPK10-2	Bra007475	1170	389	5	29400502 - 29402923
BrMPK10-3	Bra007476	1203	400	5	29404831 - 29406988
BrMPK10-4	Bra014528	1173	390	5	1229532 - 1231456
BrMPK12-1	Bra039292	1119	372	5	18731137 - 18732711
BrMPK12-2	Bra004959	1083	360	6	2659421 - 2661290
BrMPK13	Bra031597	1116	371	5	35657276 - 35659219
BrMPK15	Bra003834	1521	506	10	15271574 - 15274675
BrMPK16-1	Bra006490	1677	558	9	3763743 - 3766243
BrMPK16-2	Bra002201	1512	503	7	10936466 - 10938928
BrMPK17-1	Bra024886	1464	487	8	24106611 - 24108897
BrMPK17-2	Bra017450	1461	486	7	15494461 - 15497022
BrMPK17-3	Bra026665	1785	594	9	21239651 - 21245648
BrMPK18-1	Bra039676	1767	588	8	592117 - 594798
BrMPK18-2	Bra038128	1698	565	6	10303027 - 10305483
BrMPK19-1	Bra027317	1779	592	7	20291869 - 20294226
BrMPK19-2	Bra021573	1812	603	7	24412279 - 24414688
BrMPK20	Bra000277	1938	645	8	10294003 - 10296702
Capsella rubella					

CrMPK2	Carubv10020272m	1371	456	2	2534457 - 2536642
CrMPK3	Carubv10017466m	1113	370	5	6745397 - 6747795
CrMPK4	Carubv10003760m	1131	376	5	16292808 - 16294696
CrMPK5	Carubv10001195m	1131	376	5	13723845 - 13726117
CrMPK6	Carubv10023366m	1197	398	5	13346096 - 13350790
CrMPK7	Carubv10013994m	1107	368	2	13680760 - 13682978
CrMPK8	Carubv10008665m	1764	587	10	6263464 - 6266965
CrMPK9	Carubv10013210m	1887	628	10	6295705 - 6299966
CrMPK10	Carubv10019032m	1206	401	5	12373858 - 12376004
CrMPK11	Carubv10009488m	1116	371	5	240042 - 242239
CrMPK12	Carubv10025007m	1119	372	5	14236807 - 14238465
CrMPK13	Carubv10010840	1092	363	5	2508775 - 2510271
CrMPK14	Carubv10005075m	1116	371	1	1423095 - 1424335
CrMPK15	Carubv10021638m	1770	589	10	11270250 - 11273407
CrMPK16	Carubv10003167m	1674	557	8	6397774 - 6400238
CrMPK17	Carubv10017112m	1464	487	8	145951 - 148639
CrMPK18	Carubv10008989m	1455	484	8	18800271 - 18804171
CrMPK19	Carubv10013281m	1797	598	9	5051658 - 5057494
CrMPK20	Carubv10025156m	1821	606	9	13017052 - 13020454
Carica papaya					
CpMPK3	evm.model.supercontig_139.47	1113	370	5	358400 - 360860

CpMPK6	evm.model.supercontig_343.2	1218	405	5	5872 - 10478
CpMPK7	evm.model.supercontig_6.174	1119	372	1	1435617 - 1439341
CpMPK9	evm.model.supercontig_3.416	1659	552	10	2948541 - 2955310
CpMPK12	evm.model.supercontig_184.17	1125	374	5	162607 - 170057
CpMPK13	evm.model.supercontig_139.35	1122	373	5	214726 - 218018
CpMPK17	evm.model.supercontig_65.147	1593	530	9	1291731 – 1295805
CpMPK19	evm.TU.contig_29215.1	1794	597	8	6301 - 10777
CpMPK20	evm.model.supercontig_50.81	1893	630	9	803302 – 809482
Chlamydomonas reinhardtii					
CreinMPK4-1	Cre01.g010000.t1.2	1170	389	9	1838122 - 1842287
CreinMPK4-2	Cre12.g508900.t1.2	1128	375	7	2129460 - 2133325
CreinMPK4-3	Cre12.g509000.t1.3	1062	353	8	2119590 - 2122035
CreinMPK7	Cre13.g607300.t1.2	1302	433	10	5094976 - 5099387
CreinMPK15	Cre08.g385050.t1.3	2223	740	11	4906426 - 4913322
CreinMPK18	g18025.t1	2298	765	8	6855222 - 6862749
Citrus clememtina					
CcMPK1	Ciclev10001531m	1122	373	2	41316978 - 41320780
CcMPK3	Ciclev10028667m	1128	375	5	22568166 - 22571151
CcMPK4	Ciclev10020633m	1134	377	5	1540174 - 1544282
CcMPK6	Ciclev10020481m	1197	398	5	28402147 - 28407834

CcMPK7	Ciclev10025905m	1107	368	2	1714251 - 1717280
CcMPK9	Ciclev10007788m	1815	604	10	14383197 - 14392828
CcMPK12	Ciclev10020645m	1128	375	5	48758034 - 48761602
CcMPK13	Ciclev10028684m	1116	371	5	22715772 - 22719805
CcMPK16	Ciclev10004660m	1674	557	9	2732900 - 2738896
CcMPK17	Ciclev10018252m	1521	506	8	27774863 - 27778271
CcMPK19	Ciclev10019362m	1827	608	9	30515493 - 30520783
CcMPK20	Ciclev10030977m	1860	619	9	16129999 - 16136902
Citrus sinensis					
CsMPK1	orange1.1g017352m	1122	373	2	3212691 - 3216554
CsMPK3	orange1.1g025683m	750	249	3	1150578 - 1152598
CsMPK4	orange1.1g017083m	1134	377	5	946210 - 950342
CsMPK6	orange1.1g023609m	843	280	4	143223 - 148754
CsMPK7	orange1.1g017909m	1095	364	2	180686 - 183778
CsMPK9	orange1.1g007428m	1815	604	10	716025 - 725326
CsMPK12	orange1.1g017231m	1128	375	5	2212141 - 2215705
CsMPK13	orange1.1g042681m	1020	339	6	999867 - 1003411
CsMPK16	orange1.1g008639m	1677	558	9	1425311 - 1430726
CsMPK17	orange1.1g044076m	1521	506	8	526860 - 530539
CsMPK19	orange1.1g015019	1245	414	8	262440 - 267466
CsMPK20	orange1.1g007177m	1845	614	8	376238 - 381445

Coccomyxa subellipsoidea					
CsubMPK3	14112	1080	359	7	2065008 - 2068622
CsubMPK4	11831	1134	377	5	3252389 - 3254945
CsubMPK7	47764	1176	391	9	285901 - 290751
CsubMPK15	54284	1614	537	8	1460724 - 1464390
Cucumis sativus					
CsatMPK2	Cucsa.231940	1161	386	2	102549 - 106363
CsatMPK3	Cucsa.213350	1113	370	5	1122224 - 1126175
CsatMPK4-1	Cucsa.135720	1113	370	5	534694 - 539318
CsatMPK4-2	Cucsa.303420	1152	383	5	1363329 - 1367534
CsatMPK6	Cucsa.327650	2009	670	6	22755 - 26891
CsatMPK7	Cucsa.106400	1107	368	2	18398 - 20794
CsatMPK9-1	Cucsa.121850	1440	479	11	252941 - 256957
CsatMPK9-2	Cucsa.111480	1944	647	11	252271 - 258056
CsatMPK13	Cucsa.127090	1113	370	5	898367 - 901930
CsatMPK16	Cucsa.363560	1701	566	9	1085009 - 1091020
CsatMPK17	Cucsa.019360	1404	467	10	229293 - 235035
CsatMPK19	Cucsa.273840	1479	492	9	856821 - 861390
CsatMPK20-1	Cucsa.185440	1821	606	9	921362 - 925635
CsatMPK20-2	Cucsa.139560	1863	620	9	773251 - 778719
Eucalyptus grandis					

EgMPK1	Eucgr.F01598	1119	372	3	20289475 - 20293571
EgMPK3	Eucgr.J00966	1122	373	5	10511353 - 10514982
EgMPK4-1	Eucgr.E00103	1128	375	5	927662 - 932274
EgMPK4-2	Eucgr.E00652	1134	377	5	6173013 - 6177101
EgMPK6-1	Eucgr.G01599	1188	395	5	28429144 - 28436653
EgMPK6-2	Eucgr.L00026	771	256	4	345380 - 348888
EgMPK9-1	Eucgr.B00871	1869	622	10	10730203 - 10736384
EgMPK9-2	Eucgr.K02264	1818	605	11	29839519 - 29845047
EgMPK13	Eucgr.J02112	1161	386	5	27094672 - 27098229
EgMPK16	Eucgr.B01157	1683	560	9	18380964 - 18387552
EgMPK17	Eucgr.B02905	1521	506	10	52869167 - 52877796
EgMPK19	Eucgr.F01374	1845	614	9	17923496 - 17928482
EgMPK20	Eucgr.A01974	1857	618	9	30445600 - 30451304
Fragaria vesca					
FvMPK1	gene15192-v1.0-hybrid	1119	372	1	22508007 - 22509835
FvMPK3	gene25390-v1.0-hybrid	1239	412	6	19249762 - 19252170
FvMPK4-1	gene19238-v1.0-hybrid	1122	373	5	2788930 - 2792684
FvMPK4-2	gene31827-v1.0-hybrid	1233	410	5	1923265 - 1926740
FvMPK6	gene10128-v1.0-hybrid	1176	391	4	491878 - 494862
FvMPK7	gene14943-v1.0-hybrid	1113	370	1	8393664 - 8395106
FvMPK9	gene09401-v1.0-hybrid	2076	691	10	10150969 - 10155274

FvMPK13	gene25407-v1.0-hybrid	1851	616	10	19421394 - 19432433
FvMPK16	gene06108-v1.0-hybrid	1668	555	8	16165849 - 16169688
FvMPK19	gene27365-v1.0-hybrid	1734	577	8	24635931 - 24639525
FvMPK20	gene28706-v1.0-hybrid	2574	857	13	29782521 - 29793912
Glycine max					
GmMPK1-1	Glyma06g03270	1116	371	3	2301058 - 2303992
GmMPK1-2	Glyma04g03210	1116	371	2	2347024 - 2349849
GmMPK3-1	Glyma12g07770	1116	371	5	5380020 - 5383515
GmMPK3-2	Glyma11g15700	1116	371	5	11369716 - 11373376
GmMPK4-1	Glyma11g02420	1053	350	5	1539513 - 1542026
GmMPK4-2	Glyma08g02060	1143	380	5	1402352 - 1406155
GmMPK4-3	Glyma05g37480	1146	381	5	41066179 – 41072136
GmMPK4-4	Glyma01g43100	1128	375	5	54188362 - 54191842
GmMPK4-5	Glyma09g39190	1122	373	5	44324495 - 44330083
GmMPK4-6	Glyma07g07270	1122	373	5	5979332 - 5986189
GmMPK4-7	Glyma16g03670	1122	373	5	3084963 - 3092540
GmMPK4-8	Glyma18g47140	1122	373	5	56832304 - 56837929
GmMPK6-1	Glyma02g15690	1176	391	6	14175841 - 14181210
GmMPK6-2	Glyma07g32750	1302	433	6	37651624 - 37656682
GmMPK7-1	Glyma08g12150	1107	368	3	8838041 - 8841615
GmMPK7-2	Glyma05g28980	1107	368	2	34692976 - 34695979

GmMPK9-1	Glyma08g05700	1770	589	10	4059659 - 4064508
GmMPK9-2	Glyma05g33980	1785	594	10	38460717 - 38465803
GmMPK9-3	Glyma09g30790	1536	511	10	37564401 - 37570990
GmMPK9-4	Glyma07g11470	1524	507	10	9651174 - 9657518
GmMPK13-1	Glyma12g07850	1131	376	5	5486744 - 5490863
GmMPK13-2	Glyma11g15590	1122	373	5	11262469 - 11266621
GmMPK16-1	Glyma17g02220	1587	528	8	1412184 - 1417879
GmMPK16-2	Glyma13g28120	1692	563	9	31200711 - 31207502
GmMPK16-3	Glyma15g10940	1686	561	9	7974682 - 7981035
GmMPK19-1	Glyma13g33860	1383	460	9	35545901 - 35550612
GmMPK19-2	Glyma15g38490	1824	607	9	44920077 - 44924919
GmMPK20-1	Glyma14g03190	1836	611	9	2035902 - 2041951
GmMPK20-2	Glyma02g45630	1836	611	10	49816299 - 49823607
GmMPK20-3	Glyma08g42240	1848	615	9	42224157 - 42231045
GmMPK20-4	Glyma18g12720	1845	614	9	11933971 - 11940663
Gossipium raimondi					
GrMPK2-1	Gorai.009G199400	1119	372	2	15437996 - 15441157
GrMPK2-2	Gorai.005G109500	1119	372	2	19785322 - 19788502
GrMPK3-1	Gorai.009G104600	1131	376	5	7578096 - 7580847
GrMPK3-2	Gorai.003G139900	1128	375	5	40269900 - 40272914
GrMPK4-1	Gorai.004G159400	1113	370	6	44906698 - 44912190

GrMPK4-2	Gorai.003G155700	1125	374	5	2470910- 42474929
GrMPK4-3	Gorai.008G120100	1125	374	6	35648851 - 35653601
GrMPK4-4	Gorai.008G249800	1137	378	5	53384405 - 53387337
GrMPK4-5	Gorai.001G158800	1101	366	6	22813223 - 22817432
GrMPK4-6	Gorai.001G159000	1125	374	7	22848222 - 22853975
GrMPK6-1	Gorai.005G123100	1200	399	5	26853885 - 26858213
GrMPK6-2	Gorai.005G011100	1194	397	5	764244 - 768637
GrMPK6-3	Gorai.011G132800	1197	398	5	20168774 - 20172658
GrMPK7-1	Gorai.007G050000	1107	368	2	3517042 - 3520310
GrMPK7-2	Gorai.003G012800	1107	368	1	820961 - 823966
GrMPK7-3	Gorai.011G100600	1107	368	2	11218571 - 11221939
GrMPK7-4	Gorai.008G065400	1122	373	3	10488803 - 10490465
GrMPK9-1	Gorai.007G004400	1791	596	10	353489 - 360189
GrMPK9-2	Gorai.008G289200	1779	592	10	56399946 - 56406216
GrMPK13	Gorai.009G103800	1116	371	5	7508675 - 7511720
GrMPK16	Gorai.005G035600	1668	555	10	3385075 - 3391544
GrMPK17-1	Gorai.002G178700	1521	506	9	46384459 - 46388145
GrMPK17-2	Gorai.005G161300	1572	523	11	46634314 - 46639125
GrMPK19-1	Gorai.009G361300	1803	600	9	47602366 - 47607999
GrMPK19-2	Gorai.007G332300	1797	598	9	55489254 - 55494495
GrMPK19-3	Gorai.002G045300	1785	594	9	3780731 - 3786538

GrMPK20-1	Gorai.012G041500	1821	606	9	5215912 - 5222014
GrMPK20-2	Gorai.006G007700	1887	628	9	1589090 - 1597393
Linum usitatissimum					
LuMPK2-1	Lus10010637	1128	375	1	214630 - 216440
LuMPK2-2	Lus10033197	1128	375	1	1105749-1107600
LuMPK3-1	Lus10036136	1134	377	5	1425962 - 1427592
LuMPK3-2	Lus10018127	1134	377	5	318980 - 320595
LuMPK3-3	Lus10038472	1113	370	5	1508441 - 1511262
LuMPK3-4	Lus10023339	1137	378	5	598919-602248
LuMPK4-1	Lus10007921	1137	378	5	304241 - 306974
LuMPK4-2	Lus10024668	1167	388	5	681038 - 683039
LuMPK4-3	Lus10017518	1146	381	5	369825 - 372510
LuMPK4-4	Lus10032295	1446	481	8	583996 - 598262
LuMPK4-5	Lus10036384	1140	379	5	390221 - 392741
LuMPK4-6	Lus10028765	1146	381	5	138953 - 141784
LuMPK6-1	Lus10027091	1248	415	5	670213 - 678964
LuMPK6-2	Lus10008339	1248	415	5	169578 - 173692
LuMPK7-1	Lus10014283	1104	367	1	209111 - 210634
LuMPK7-2	Lus10025986	1110	369	1	189862 - 191292
LuMPK9-1	Lus10027248	1401	466	9	479198 - 482471
LuMPK9-2	Lus10038956	1503	500	10	1006720 - 1009547

LuMPK16-2	Lus10021945	1764	587	8	697707 - 700450
LuMPK17-1	Lus10021784	1533	510	8	209422 - 212471
LuMPK17-2	Lus10034601	1410	469	8	892359 - 895141
LuMPK16-1	Lus10041234	1683	560	9	154329-1546668
LuMPK18-1	Lus10005568	1851	616	9	116964 - 120611
LuMPK18-2	Lus10013702	1842	613	9	119756 - 123446
Malus domestica					
MdMPK1-1	MDP0000165532	1102	372	1	11220 - 13007
MdMPK1-2	MDP0000210110	1119	372	1	21620 - 23316
MdMPK1-3	MDP0000128473	1102	372	1	3956 - 15742
MddPK3-1	MDP0000321850	1103	370	5	2814 - 4826
MdMPK3-2	MDP0000237742	1110	370	5	2372 - 4411
MdMPK3-3	MDP0000199036	1103	370	5	25333 - 27345
MdMPK4-1	MDP0000766240	1134	377	5	19367 - 22897
MdMPK4-2	MDP0000251955	1105	373	5	2529 - 6362
MdMPK4-3	MDP0000321746	2335	798	7	4579 - 13897
MdMPK4-4	MDP0000326020	1097	374	5	2517 - 6540
MdMPK6-1	MDP0000340624	1209	407	4	18566 - 21753
MdMPK6-2	MDP0000321308	1173	403	4	5057 - 8429
MdMPK7-1	MDP0000807889	1135	378	1	21141 - 22624
MdMPK7-2	MDP0000826016	1130	378	1	8163 - 9648

MdMPK9	MDP0000294142	1902	634	10	7823 - 11834
MdMdK13-1	MDP0000189383	1540	515	3	1798 - 3572
MdMPK13-2	MDP0000173178	1541	515	3	525 - 2299
MdMPK13-3	MDP0000593502	1118	374	5	7934 - 10412
MdMPK13-4	MDP0000422421	1444	484	7	15064 - 20165
MdMPK16-1	MDP0000879089	1746	588	9	5769 - 9303
MdMPK16-2	MDP0000170804	1993	674	11	5246 - 10064
MdMPK17	MDP0000277562	1739	583	10	10293 - 13705
MdMPK19-1	MDP0000169216	1881	630	9	9941 - 14437
MdMPK19-2	MDP0000121116	1818	608	9	4663 - 9094
MdMPK19-3	MDP0000195781	1844	616	9	15074 - 19363
MdMPK19-4	MDP0000233021	1870	627	9	56573 - 61036
MdMPK20-1	MDP0000188369	1817	608	9	36671 - 40209
MdMPK20-2	MDP0000250639	2578	865	14	494 - 6903
Manihot esculenta					
MeMPK1	cassava4.1_009963m	1119	372	2	1005650 - 1009694
MeMPK2	cassava4.1_009941m	1119	372	2	173215 - 177559
MeMPK3	cassava4.1_010219m	1098	365	5	136124 - 139862
MeMPK4-1	cassava4.1_009716m	1140	379	5	125152 - 128569
MeMPK4-2	cassava4.1_009399m	1170	389	5	565579 - 569442
MeMPK4-3	cassava4.1_010005m	1113	370	5	1234141 - 1239535

MeMPK4-4	cassava4.1_009957m	1119	372	5	728311 - 732627
MeMPK6	cassava4.1_008933m	1215	404	4	25882 - 35185
MeMPK9-1	cassava4.1_004009m	1803	600	9	325016 - 331447
MeMPK9-2	cassava4.1_003987m	1809	602	10	299 - 13438
MeMPK13	cassava4.1_009999m	1116	371	5	457067 - 459571
MeMPK16-1	cassava4.1_004681m	1689	562	9	2062249 - 2068343
MeMPK16-2	cassava4.1_004688m	1689	562	9	37373 - 144762
MeMPK17-1	cassava4.1_006340m	1476	491	8	60841 - 65239
MeMPK17-2	cassava4.1_006140m	1497	498	9	372621 - 378417
MeMPK19	cassava4.1_005598m	1560	519	8	125589 - 129978
MeMPK20	cassava4.1_004025m	1803	600	8	733220 - 742212
Medicago truncatula					
MtMPK3	Medtr4g061130	1071	356	6	8965437 - 18968342
MtMPK4-1	Medtr7g038040	1116	371	5	0960093 - 10965221
MtMPK4-2	Medtr7g078690	1119	372	5	22011328 - 22015936
MtMPK4-3	Medtr5g010030	1128	375	5	2369912 - 2371946
MtMPK6	Medtr4g087620	1164	387	5	30356106 - 30362314
MtMPK7-1	Medtr3g060350	837	278	5	18560233 - 18561834
MtMPK7-2	Medtr3g060390	1005	334	3	18571187 - 18572652
MtMPK7-3	Medtr3g060330	1107	368	1	18555646 - 18556970
MtMPK7-4	Medtr8g086000	1266	421	3	23636927 - 23639616 :

MtMPK7-5	Medtr8g086010	1269	422	6	23641382 - 23644784
MtMPK9	Medtr8g106960	1848	615	10	32068746 - 32073632
MtMPK13	Medtr4g061320	1125	374	5	19055628 - 19059692
MtMPK16	Medtr2g021330	1695	564	9	7209119 - 7215501
MtMPK19	AC235677_37	1827	608	9	144610 - 149107
MtMPK20-1	Medtr3g089510	1845	614	9	30090068 - 30096709
MtMPK20-2	Medtr5g091680	1509	502	10	38953398 - 38958773
MtMPK20-3	AC225528_53	1824	607	8	224474 - 228900
Micromonas pusila CCMP1545					
MpMPK2	203785	1125	374	1	733229 - 734490
MpMPK4	27380	1281	426	4	973110 - 975330
MpMPK13	14294	1143	380	9	84807 - 486526
MpMPK15	44271	1089	362	2	357924 - 360582
Mimulus guttatus					
MgMPK1	mgv1a008410m	1125	374	3	181405 - 184207
MgMPK4-1	mgv1a009790m	996	331	4	1381166 - 1385339
MgMPK4-2	mgv1a008501m	1116	371	5	328618 - 332328
MgMPK9	mgv1a005228m	1479	492	11	985602 - 989271
MgMPK17	mgv1a005103m	1494	497	10	160355 - 164343
MgMPK20	mgv1a003659m	1716	571	8	128616 - 133220
Oryza sativa					

OsMPK3	LOC_Os03g17700	1110	370	5	9850473-9847700
OsMPK4-1	LOC_Os10g38950	1131	377	5	20756089-2076021
OsMPK4-2	LOC_Os05g05160	1629	543	9	2511542-2518105
OsMPK6	LOC_Os06g06090	1197	399	5	2813004-2806543
OsMPK7	LOC_Os06g48590	1110	370	2	29398207-29402539
OsMPK14	LOC_Os02g05480	1113	371	2	2646360-2642977
OsMPK16-1	LOC_Os11g17080	1497	499	9	9471521-9479355
OsMPK16-2	LOC_Os08g06060	1185	395	5	3307520-3310590
OsMPK17-1	LOC_Os06g49430	1743	581	10	29949829-29954874
OsMPK17-2	LOC_Os02g04230	1521	507	10	1857067-1850954
OsMPK20-1	LOC_Os01g43910	1836	612	9	25158379-25152102
OsMPK20-2	LOC_Os05g50560	1050	350	8	28989786-28995672
OsMPK20-3	LOC_Os06g26340	1713	571	9	15423582-15414829
OsMPK20-4	LOC_Os01g47530	1773	591	9	27171287-27178431
OsMPK20-5	LOC_Os05g49140	1779	593	9	28194025-28188762
OsMPK21-1	LOC_Os05g50120	1749	583	10	28721012-28726522
OsMPK21-2	LOC_Os01g45620	1506	502	9	25922978-25917623
Ostreococcus lucimarinus					
OlMPK6	C_Chr_9000126	1218	405	1	599832 - 601063
OlMPK7	e_gwEuk.9.247	1350	449	0	61142 - 62491
OlMPK9	gwEuk.8.109.1 gw	1161	387	0	65307 - 66467

Panicum virgatum					
PvMPK3-1	Pavirv00035754m	1113	371	5	sg0.contig96967: 2 - 3391
PvMPK3-2	Pavirv00022561m	975	324	5	sg0.contig03041: 11047 - 12974
PvMPK4-1	Pavirv00033923m	1128	375	6	sg0.contig26991: 2154 - 6217
PvMPK4-2	Pavirv00003084m	1122	373	6	sg0.contig34630: 989 - 5126
PvMPK4-3	Pavirv00026757m	1170	389	5	sg0.contig24978: 4585 – 8109
PvMPK4-4	Pavirv00067074m	822	273	3	sg0.contig203924: 226 - 1983
PvMPK6-1	Pavirv00041892m	1188	395	5	sg0.contig17157: 4342 - 9992
PvMPK6-2	Pavirv00064097m	1191	396	5	sg0.contig22718: 254 - 5672
PvMPK7-1	Pavirv00007881m	1110	369	1	sg0.contig45220: 2461 - 5479
PvMPK7-2	Pavirv00059104m	543	180	0	sg0.contig200225: 680 - 1697
PvMPK14-1	Pavirv00021718m	1113	370	1	sg0.contig143589: 55 - 2478
PvMPK14-2	Pavirv00050004m	1113	370	1	sg0.contig74029: 1121 - 3866
PvMPK16	Pavirv00037141m	1638	545	9	sg0.contig00168: 2095 - 8882
PvMPK17-1	Pavirv00037180m	1725	574	10	sg0.contig00053: 10482 - 15810
PvMPK17-2	Pavirv00047952m	1386	461	11	sg0.contig16313: 1731 - 8807
PvMPK17-3	Pavirv00045770m	1578	525	10	sg0.contig13237: 2021 - 9223
PvMPK17-4	Pavirv00062557m	1725	574	10	sg0.contig01366: 4478 - 9813
PvMPK20-1	Pavirv00037472m	1830	609	9	sg0.contig00626: 42 - 6071
PvMPK20-2	Pavirv00069001m	1893	630	9	sg0.contig08205: 2187 - 6023
PvMPK20-3	Pavirv00036824m	1830	609	9	sg0.contig00165: 8778 - 15155

PvMPK20-4	Pavirv00038393m	1767	588	9	sg0.contig00299: 308 - 5821
PvMPK20-5	Pavirv00029083m	1749	582	9	sg0.contig05778: 7702 - 12901
PvMPK20-6	Pavirv00062029m	1776	591	9	sg0.contig01695: 1854 - 6893
PvMPK20-7	Pavirv00061344m	1920	639	8	sg0.contig01329: 13727 - 18267
PvMPK21-1	Pavirv00045069m	1545	514	10	sg0.contig26195: 3433 - 7879
PvMPK21-2	Pavirv00070777m	1419	473	7	sg0.contig33407: 2406 - 6832
PvMPK21-3	Pavirv00054256m	1509	502	9	sg0.contig21365: 1457 - 6740
Phaseolus vulgaris					
PvulMPK1	Phvul.009G061000	1116	371	2	10784536 - 10787306
PvulMPK3	Phvul.011G071400	1116	371	5	6384955 - 6388514
PvulMPK4-1	Phvul.010G102800	1122	373	5	36007058 - 36012871
PvulMPK4-2	Phvul.002G292400	1146	381	6	45574376 - 45577692
PvulMPK4-3	Phvul.002G159500	1119	372	5	30143791 - 30147397
PvulMPK6	Phvul.003G059500	1185	394	6	7997521 - 8002353
PvulMPK7	Phvul.002G237500	1107	368	3	40326916 - 40330333
PvulMPK9	Phvul.004G151200	1518	505	10	43209532 - 43215372
PvulMPK13	Phvul.011G070800	1365	454	5	6239531 - 6243990
PvulMPK16-1	Phvul.003G095900	1710	569	10	21721337 - 21727877
PvulMPK16-2	Phvul.006G155300	1692	563	9	26823486 - 26829918
PvulMPK19	Phvul.005G069800	1821	606	9	11374844 - 11379538
PvulMPK20-1	Phvul.008G191500	1842	613	9	49722993 - 49729169

PvulMPK20-2	Phvul.006G033600	1809	602	9	13767639 - 13774715
Physcomitrella patens V 1.3					
PpMPK1	Pp1s207_63V6	1107	368	1	450742 - 452799
PpMPK2	Pp1s138_117V6	1107	368	1	652015 - 653435
PpMPK4-1	Pp1s99_26V6	1947	648	7	218432 - 222835
PpMPK4-2	Pp1s59_325V6	1143	380	4	1663035 - 1666288
PpMPK4-3	Pp1s149_39V6	1128	375	6	42484 - 146003
PpMPK4-4	Pp1s29_285V6	2052	683	7	2013329 - 2017763
PpMPK16-1	Pp1s80_71V6	1599	532	10	438428 - 443542
PpMPK16-2	Pp1s87_157V6	1602	533	10	1153001 - 1157659
Picea abies					
PaMPK1	MA_10428223g0010	1107	368	1	MA_10428223:2779…18459
PaMPK2	MA_9261976g0010	540	179	0	MA_9261976:1883…2462
PaMPK3	MA_160202g0010	540	179	0	MA_160202:6574…7153
PaMPK4	MA_10437018g0010	1272	423	6	MA_104718:28709…43654
PaMPK5	MA_42176g0010	1215	404	1	MA_42176:27987…29858
PaMPK6	MA_10437020g0010	1410	469	10	MA10437020:12198…31995
PaMPK7-1	MA_8212817g0010	543	180	0	MA_8212817:860…1442
PaMPK7-2	MA_60199g0010	1248	145	1	MA_60199:12944…15019
PaMPK8	MA_10427605g0010	651	216	2	MA_10427605:3195…5258
PaMPK10	MA_117156g0010	1095	364	1	MA_117156:4558…6750

PaMPK11	MA_10432928g0010	873	290	4	MA_10432928:1859…2423
PaMPK14	MA_42176g0010	1215	404	1	MA_42176:27987…29858
PaMPK16	MA_91728g0010	2250	749	12	MA_91728:13754…32130
PaMPK20	MA_8212817g0010	543	180	0	MA_8212817:860…1442
Populus trichocarpa					
PtMPK1	Potri.002G032100	1119	372	3	2054473 - 2058388
PtMPK2	Potri.005G231100	1146	381	2	23975593 - 23979241
PtMPK3-1	Potri.009G066100	1116	371	5	6652439 - 6656080
PtMPK3-2	Potri.001G271700	1113	370	5	27917216 - 27923982
PtMPK4-1	Potri.002G162500	1119	372	5	12265644 - 12270287
PtMPK4-2	Potri.014G088500	1119	372	5	6980125 - 6985518
PtMPK6-1	Potri.017G010200	1200	399	5	871837 - 876910
PtMPK6-2	Potri.007G139800	1197	398	5	15087009 - 15092165
PtMPK7-1	Potri.007G020100	1107	368	2	1509842 - 1512537
PtMPK7-2	Potri.005G119500	1128	375	2	9259211 - 9262862
PtMPK9-1	Potri.015G040300	1818	605	11	722584 - 3731025
PtMPK9-2	Potri.012G048600	1779	592	10	4553202 - 4559544
PtMPK12	Potri.003G131800	1101	366	5	5129437 - 15133928
PtMPK16-1	Potri.010G029700	1689	562	9	4284285 - 4290019
PtMPK16-2	Potri.008G200800	1689	562	9	14103851 - 14109874
PtMPK17-1	Potri.010G112200	1521	506	9	13079177 - 13083275

PtMPK17-2	Potri.008G130000	1458	485	8	8516477 - 8520064
PtMPK19-1	Potri.011G102500	1791	596	9	12509073 - 12515342
PtMPK19-2	Potri.001G381300	1833	610	9	9725628 - 39731752
PtMPK20-1	Potri.002G059900	1866	621	9	4075718 - 4083365
PtMPK20-2	Potri.005G201800	1872	623	9	21696792 - 21703421
Prunus persica					
PperMPK1	ppa007332m	1119	372	1	33034929 - 33037178
PperMPK3	ppa007370m	1113	370	5	6284824 - 6287237
PperMPK4-1	ppa007254m	1131	376	5	11191348 - 11195209
PperMPK4-2	ppa007306m	1122	373	5	18524622 - 18529615
PperMPK6	ppa006536m	1124	407	4	22439450 - 22442626
PperMPK7	ppa007418m	1107	368	2	17083831 - 17086605
PperMPK9-1	ppa002837m	1887	628	10	17650631 - 17654904
PperMPK9-2	ppa003297m	1764	587	10	30055217 - 30059887
PperMPK13	ppa007376m	1113	370	5	6169277 - 6172169
PperMPK16	ppa005596m	1359	452	8	1242388 - 11246409
PperMPK19	ppa003651m	1680	559	8	8848946 - 8853887
PperMPK20	ppa002953m	1857	618	9	13723138 - 13728692
Ricinus communis					
RcMPK2	30170.t000201	1119	372	1	2765978 - 2768635
RcMPK3	29688.t000016	843	280	3	108064 - 109922

RcMPK4-1	30174.t000044	1113	370	5	1926472 -1930746
RcMPK4-2	30190.t000501	1164	387	5	2908926 - 2911847
RcMPK6	29747.t000005	1209	402	5	25988 - 33431
RcMPK7	29634.t000023	1107	368	1	159630 - 161923
RcMPK9	28097.t000002	1842	613	10	14778 - 22904
RcMPK13	28752.t000009	1119	372	5	60782 - 63243
RcMPK15	29816.t000019	1512	503	8	207146 - 211249
RcMPK16	29726.t000028	1695	564	9	194650 - 199776
RcMPK19	29989.t000008	1821	606	8	53154-57320
RcMPK20	29682.t000030	1884	627	9	207494 - 215361
Selaginella moellendorffii					
SmMPK1	443152	1125	374	1	746929 - 748943
SmMPK4	105143	1125	374	5	1754333 - 1755803
SmMPK7	75282	1113	370	1	2633329 - 2634498
SmMPK10	82767	1143	380	14	056461 - 3058391
SmMPK16-1	74687	1425	474	7	4873903 - 4875721
SmMPK16-2	97841	1227	408	7	687993 - 689791
Setaria italica					
SiMPK3	Si036218m	1128	375	5	49447344 - 49452685
SiMPK4-1	Si036240m	1119	372	6	40427293 - 40431206
SiMPK4-2	Si013899m	1173	390	5	4549915 - 4553473

SiMPK6	Si006611m	1182	393	5	5382023 - 5387940
SiMPK7	Si006708m	1110	369	3	36733990 - 36738213
SiMPK14	Si017554m	1113	370	2	7886461 - 7890224
SiMPK16-1	Si021645m	1674	557	9	3700371 - 3706333
SiMPK16-2	Si026197m	1608	535	8	14013196 - 14019473
SiMPK17-1	Si006144m	1725	574	10	39101417 - 39106533
SiMPK17-2	Si016957m	1521	506	10	8369629 - 8375826
SiMPK20-1	Si000725m	1836	611	9	29847140 - 29853255
SiMPK20-2	Si021664m	1650	549	8	9128086 - 9133123
SiMPK20-3	Si021560m	1776	591	9	10338871 - 10343433
SiMPK20-4	Si000788m	1770	589	9	32399390 - 32405601
SiMPK21-1	Si021565m	1773	590	10	9482394 - 9487115
SiMPK21-2	Si004793m	1443	480	8	30469015 - 30473875
Solanum lycopersicum					
SlMPK1	Solyc04g080730	1119	372	3	62421080 - 62429518
SlMPK3	Solyc06g005170	1122	373	5	192235 - 195321
SlMPK4-1	Solyc05g049970	1131	376	5	58964608 - 58969530
SlMPK4-2	Solyc01g094960	1122	373	5	78118441 - 78124258
SlMPK5	Solyc08g081490	1140	379	5	61696989 - 61700296
SlMPK6-1	Solyc08g014420	1185	394	5	4354487 - 4377242
SlMPK6-2	Solyc12g019460	1191	396	5	10385358 - 10395971

SlMPK7	Solyc02g084870	1113	370	2	42513397 - 42516636
SlMPK9-1	Solyc06g068990	1806	601	10	39197548 - 39203557
SlMPK9-2	Solyc12g040680	1728	575	10	40280910 - 40287741
SlMPK13	Solyc11g072630	1119	372	5	52945837 - 52949594
SlMPK15-1	Solyc04g007710	1539	512	11	1389688 - 1396477
SlMPK15-2	Solyc05g008020	1542	513	10	2439987 - 2443704
SlMPK16	Solyc01g080240	1698	565	9	71971471 - 71978931
SlMPK19-1	Solyc10g007500	1782	593	9	1832683 - 1838376
SlMPK19-2	Solyc07g062080	1803	600	9	62129987 - 62135063
SlMPK20	Solyc07g056350	1866	621	9	61549395 - 61555184
Solanum tubersum					
StMPK1	PGSC0003DMG400003700	1119	372	1	62260111 - 62266969
StMPK4-1	PGSC0003DMG400021649	1131	376	5	53290786 - 53297704
StMPK4-2	PGSC0003DMG400012188	1140	379	5	42989687 - 42994389
StMPK4-3	PGSC0003DMG401000057	1122	373	5	78685081 - 78691827
StMPK7	PGSC0003DMG400003528	1113	370	1	62167610 - 62171410
StMPK9	PGSC0003DMG402028796	1818	605	10	45661534 - 45668263
StMPK15-1	PGSC0003DMG400005955	1539	512	10	837725 - 846119
StMPK15-2	PGSC0003DMG400030492	1536	511	9	4150589 - 4153603
StMPK16	PGSC0003DMG400004357	1698	565	9	70758677 - 70766639
StMPK19-1	PGSC0003DMG400021253	1782	593	9	2329483 - 2335206

StMPK19-2	PGSC0003DMG400007058	1812	603	9	49619682 - 49625485
StMPK20	PGSC0003DMG400017345	1842	613	9	47770409 – 47776129
Sorghum bicolor					
SbMPK3	Sb01g038750	1125	374	5	62232922 - 62237741
SbMPK4-1	Sb01g030680	1134	377	6	53254212 - 53258199
SbMPK4-2	Sb07g003810	1167	388	5	4671118 - 4674850
SbMPK6	Sb10g003810	1209	402	5	3303588 - 3309075
SbMPK7	Sb10g028780	1110	369	2	58634496 - 58638900
SbMPK14	Sb04g003480	1113	370	2	3325168 - 3329588
SbMPK16-1	Sb05g010000	1233	410	7	20127500 - 20139007
SbMPK16-2	Sb09g003280	1671	556	9	3672016 - 3677797
SbMPK17-1	Sb10g029400	1725	574	10	59244743 - 59250233
SbMPK17-2	Sb04g002830	1473	490	9	2642929 - 2647052
SbMPK20-1	Sb03g028740	1842	613	9	56775084 - 56782423
SbMPK20-2	Sb09g029720	1797	598	9	58361463 - 58366568
SbMPK20-3	Sb09g028690	1773	590	9	57534040 - 57539411
SbMPK20-5	Sb03g030450	1743	580	9	58695454 - 58699766
SbMPK21-1	Sb09g029370	1779	592	10	58049073 - 58054449
SbMPK21-2	Sb03g029340	1287	428	9	57485238 - 57490269
Thellungiella halophila					
ThMPK2	Thhalv10023530m	1122	373	2	5934502 - 5936794

ThMPK3	Thhalv10002582m	1110	369	5	9818183 - 9820077
ThMPK4	Thhalv10028738m	1128	375	5	326832 - 329438
ThMPK5	Thhalv10028737m	1131	376	6	4670375 - 4672735
ThMPK6	Thhalv10001439m	1365	454	5	210280 - 212837
ThMPK7	Thhalv10025533m	1092	363	1	1530361 - 1531533
ThMPK8	Thhalv10007165m	1764	587	10	9282783 - 9286295
ThMPK9	Thhalv10020306m	1863	620	10	3132757 - 3136748
ThMPK10	Thhalv10006460m	1209	402	5	1310371 - 1312353
ThMPK12	Thhalv10001502m	1119	372	5	089228 - 1090978
ThMPK13	Thhalv10008001m	1092	363	5	3161397 - 13163064
ThMPK16	Thhalv10013120m	1704	567	9	6495260 - 6498847
ThMPK17	Thhalv10000832m	1731	576	10	2635626-2639437
ThMPK18	Thhalv10011329m	1815	604	9	1067721 - 1071433
ThMPK19	Thhalv10020339m	1797	598	9	4440967 - 4444726
ThMPK20	Thhalv10016406m	1806	601	9	11795846 - 11800503
Theobroma cacao					
TcMPK1	Thecc1EG034103	1119	372	2	1449491 - 1452634
TcMPK3	Thecc1EG042004	1128	375	5	40556868 - 40559705
TcMPK4-1	Thecc1EG005280	1125	374	6	35900758 - 35905389
TcMPK4-2	Thecc1EG016328	1125	374	5	32389009 - 32393572
TcMPK6	Thecc1EG019832	1284	427	6	25226327 - 25232768

TcMPK7	Thecc1EG000305	1011	336	3	1253856 - 1257638
TcMPK9	Thecc1EG012710	1902	633	11	5388005 - 5396596
TcMPK13	Thecc1EG041990	1116	371	5	40474759 - 40477881
TcMPK16	Thecc1EG020486	1692	563	9	28783304 - 28789517
TcMPK17	Thecc1EG010874	1488	495	13	36213087 - 36218810
TcMPK19	Thecc1EG032167	1806	601	10	9270290 - 9278040
TcMPK20	Thecc1EG001868	1863	620	10	9896701 - 9905061
Vitis venifera					
VvMPK1	GSVIVG01009766001	588	195	4	11125765 - 11129338
VvMPK3	GSVIVG01025105001	990	329	5	4432854 - 4436338
VvMPK4-1	GSVIVG01019406001	1128	375	5	380310 - 386888
VvMPK4-2	GSVIVG01026984001	1128	375	6	18821560 - 18826926
VvMPK6	GSVIVG01038192001	993	330	6	24220238 - 24241107
VvMPK7	GSVIVG01018883001	2310	769	9	18974001 - 19005635
VvMPK9-1	GSVIVG01008408001	1806	601	10	2368190 - 2377747
VvMPK9-2	GSVIVG01011749001	1842	613	10	4565334 - 4574753
VvMPK13	GSVIVG01025091001	1116	371	5	4580755 - 4584961
VvMPK16	GSVIVG01017873001	1797	598	9	224299 – 234190
VvMPK19	GSVIVG01014081001	1797	598	9	224299 - 234190
VvMPK20	GSVIVG01000784001	1518	505	10	124452 - 133238
Volvox carteri					

VcMPK4-1	Vocar20007163m	1194	397	9	370334 - 376278
VcMPK4-2	Vocar20000906m	1146	381	6	1657345 - 1659940
VcMPK5	Vocar20007415m	1293	430	8	3065894 - 3069287
VcMPK9	Vocar20004345m	1401	466	7	728757 - 734075
VcMPK20	Vocar20011355m.g	1935	644	7	1821090 - 1824961
Zea mays					
ZmMPK3-1	GRMZM2G053987	966	321	5	138430178 - 138436051
ZmMPK3-2	GRMZM2G017792	1131	376	4	43685604 - 43688500
ZmMPK4-1	GRMZM2G127141	1119	372	7	125125195 - 125130193
ZmMPK4-2	GRMZM2G123886	1176	391	5	79135496 - 79138192
ZmMPK6-1	GRMZM2G002100	1197	398	6	4598823- 74604163
ZmMPK6-2	GRMZM2G020216	1197	398	5	18872654 - 18878542
ZmMPK7	GRMZM2G048455	1110	369	2	56014394 - 56019719
ZmMPK14	GRMZM2G062914	1113	370	2	79862405 - 79867873
ZmMPK16	GRMZM2G089484	1674	557	9	88681122 - 88686973
ZmMPK17-1	GRMZM2G306028	1500	499	10	86113653 - 86117836
ZmMPK17-2	GRMZM2G374088	1476	491	10	239429006 - 239435447
ZmMPK17-3	GRMZM2G135904	867	289	7	59035360 - 59039091
ZmMPK20-1	GRMZM2G131334	1452	483	8	145841979 - 145848016
ZmMPK20-2	GRMZM2G163861	1809	602	9	166818256 - 166823414
ZmMPK20-4	GRMZM2G122335	1767	588	9	211128499 - 211135671

ZmMPK20-5	GRMZM2G007848	1770	589	9	149031928 - 149038235
ZmMPK20-6	GRMZM2G034052	1740	579	9	70550243 - 70553568
ZmMPK21-1	GRMZM2G062761	1806	601	10	165947457 - 165953178
ZmMPK21-2	GRMZM2G375975	1470	489	9	146830973 - 146836666
