# Supplementary material for: Identification of new members of the MAPK gene family in plants shows diverse conserved domains and novel activation loop variants
Source: BMC Genomics. 2015 Feb 6;16(1):58. doi: 10.1186/s12864-015-1244-7 (PMC4363184; doi:10.1186/s12864-015-1244-7)
Supplement: Additional file 2: — Additional data file showing the MAPK gene name, molecular weight (in kDa) and predicted isoelectric (pI) point. [file 12864_2015_1244_MOESM2_ESM.pdf]

Additional data file 2

Additional data file representing MAPK gene name, molecular weight (in kDa) and their isoelectric points.

Gene Name	Locus ID	Mol. Weight in kDa	Isoelectric point (pI)
Acquilegia coerulea			
AcMPK1	Aquca_007_00283	42.407	6.66
AcMPK2-1	Aquca_005_00492	42.215	7.33
AcMPK2-2	Aquca_010_00559	42.196	7.16
AcMPK3-1	Aquca_027_00128	43.584	5.73
AcMPK3-2	Aquca_046_00022	42.324	6.43
AcMPK4	Aquca_038_00076	53.129	8.70
AcMPK6	Aquca_030_00253	45.997	5.64
AcMPK9	Aquca_041_00181	70.815	7.66
AcMPK13	Aquca_027_00155	42.316	5.12
AcMPK20	Aquca_022_00063	70.544	9.12
Arabidopsis thaliana			
AtMPK1	At1g10210	42.644	6.98
AtMPK2	At1g59580	43.124	6.66
AtMPK3	At3g45640	42.716	5.98
AtMPK4	At4g01370	42.851	6.11
AtMPK5	At4g11330	43.207	5.92
AtMPK6	At2g43790	45.057	5.48

AtMPK7	At2g18170	42.298	7.30
AtMPK8	At1g18150	66.231	6.54
AtMPK9	At3g18040	58.394	8.41
AtMPK10	At3g59790	45.174	5.04
AtMPK11	At1g01560	31.596	7.28
AtMPK12	At2g46070	42.474	7.94
AtMPK13	At1g07880	29.453	5.70
AtMPK14	At4g36450	41.975	6.71
AtMPK15	At1g73670	65.250	8.52
AtMPK16	At5g19010	64.911	8.79
AtMPK17	At2g01450	55.493	6.93
AtMPK18	At1g53510	69.351	9.17
AtMPK19	At3g14720	67.407	9.11
AtMPK20	At2g42880	68.774	9.33
Brachypodium distachyon			
BdMPK3	Bradi1g65810	42.865	5.76
BdMPK4-1	Bradi3g32000	42.756	6.17
BdMPK4-2	Bradi3g16560	43.955	6.71
BdMPK6	Bradi1g49100	44.200	5.83
BdMPK7-1	Bradi1g34030	47.110	8.21
BdMPK7-2	Bradi4g24912	42.368	7.09

BdMPK14	Bradi3g03780	42.315	6.98
BdMPK16	Bradi2g36470	61.602	8.57
BdMPK17	Bradi1g34700	65.491	7.27
BdMPK20-1	Bradi2g44350	69.953	9.00
BdMPK20-2	Bradi2g15317	66.747	9.04
BdMPK20-3	Bradi1g41780	48.623	8.54
BdMPK20-4	Bradi2g45870	66.389	9.33
BdMPK20-5	Bradi2g16337	66.407	9.28
BdMPK21-1	Bradi2g15620	67.136	7.09
BdMPK21-2	Bradi2g45010	66.406	8.91
Brassica rapa			
BrMPK1	Bra019955	42.523	7.14
BrMPK2	Bra035437	42.576	6.66
BrMPK3	Bra038281	38.274	5.77
BrMPK4	Bra000955	42.529	6.25
BrMPK5	Bra035233	52.859	5.61
BrMPK6-1	Bra000326	44.884	5.39
BrMPK6-2	Bra004784	44.879	5.48
BrMPK7-1	Bra037234	42.278	7.52
BrMPK7-2	Bra039629	42.276	7.11
BrMPK8-1	Bra031017	59.799	7.33

BrMPK8-2	Bra025929	65.497	6.60
BrMPK9	Bra022276	68.198	7.56
BrMPK10-1	Bra014527	94.809	5.80
BrMPK10-2	Bra007475	44.361	6.05
BrMPK10-3	Bra007476	45.706	6.70
BrMPK10-4	Bra014528	44.743	5.49
BrMPK12-1	Bra039292	42.623	7.91
BrMPK12-2	Bra004959	41.261	7.95
BrMPK13	Bra031597	43.047	5.13
BrMPK15	Bra003834	57.488	7.61
BrMPK16-1	Bra006490	64.059	8.63
BrMPK16-2	Bra002201	57.760	8.50
BrMPK17-1	Bra024886	55.641	6.98
BrMPK17-2	Bra017450	55.373	7.70
BrMPK17-3	Bra026665	67.828	6.51
BrMPK18-1	Bra039676	66.242	9.20
BrMPK18-2	Bra038128	63.905	9.10
BrMPK19-1	Bra027317	66.765	9.12
BrMPK19-2	Bra021573	67.513	9.31
BrMPK20	Bra000277	72.995	8.62
Capsella rubella			

CrMPK2	Carubv10020272m	52.471	7.33
CrMPK3	Carubv10017466m	42.587	5.86
CrMPK4	Carubv10003760m	43.060	6.25
CrMPK5	Carubv10001195m	43.178	5.61
CrMPK6	Carubv10023366m	45.356	5.56
CrMPK7	Carubv10013994m	42.263	7.07
CrMPK8	Carubv10008665m	66.076	6.65
CrMPK9	Carubv10013210m	71.800	7.21
CrMPK10	Carubv10019032m	45.696	5.14
CrMPK11	Carubv10009488m	42.531	6.54
CrMPK12	Carubv10025007m	42.515	7.94
CrMPK13	Carubv10010840	42.177	5.19
CrMPK14	Carubv10005075m	43.096	8.04
CrMPK15	Carubv10021638m	66.671	7.13
CrMPK16	Carubv10003167m	63.712	8.69
CrMPK17	Carubv10017112m	55.594	6.86
CrMPK18	Carubv10008989m	55.208	9.24
CrMPK19	Carubv10013281m	67.372	9.11
CrMPK20	Carubv10025156m	68.755	9.32
Carica papaya			
CpMPK3	evm.model.supercontig_139.47	42.667	5.96

CpMPK6	evm.model.supercontig_343.2	46.075	5.56
CpMPK7	evm.model.supercontig_6.174	42.863	7.68
CpMPK9	evm.model.supercontig_3.416	62.359	8.05
CpMPK12	evm.model.supercontig_184.17	43.067	6.71
CpMPK13	evm.model.supercontig_139.35	42.842	5.17
CpMPK17	evm.model.supercontig_65.147	61.227	8.76
CpMPK19	evm.TU.contig_29215.1	67.612	9.18
CpMPK20	evm.model.supercontig_50.81	71.762	8.92
Chlamydomonas reinhardtii			
CreinMPK4-1	Cre01.g010000.t1.2	43.963	6.04
CreinMPK4-2	Cre12.g508900.t1.2	42.528	7.80
CreinMPK4-3	Cre12.g509000.t1.3	39.754	8.59
CreinMPK7	Cre13.g607300.t1.2	48.546	8.73
CreinMPK15	Cre08.g385050.t1.3	79.024	8.43
CreinMPK18	g18025.t1	81.218	7.55
Citrus clementina			
CcMPK1	Ciclev10001531m	42.847	7.28
CcMPK3	Ciclev10028667m	42.836	5.54
CcMPK4	Ciclev10020633m	43.084	6.98
CcMPK6	Ciclev10020481m	45.111	5.56
CcMPK7	Ciclev10025905m	42.322	7.89

CcMPK9	Ciclev10007788m	68.586	7.50
CcMPK12	Ciclev10020645m	43.161	6.75
CcMPK13	Ciclev10028684m	42.954	5.50
CcMPK16	Ciclev10004660m	63.250	8.89
CcMPK17	Ciclev10018252m	58.508	7.76
CcMPK19	Ciclev10019362m	69.155	8.99
CcMPK20	Ciclev10030977m	70.689	9.06
Citrus sinensis			
CsMPK1	orange1.1g017352m	42.843	7.09
CsMPK3	orange1.1g025683m	28.863	5.24
CsMPK4	orange1.1g017083m	43.084	6.98
CsMPK6	orange1.1g023609m	32.603	5.21
CsMPK7	orange1.1g017909m	41.934	7.89
CsMPK9	orange1.1g007428m	58.478	7.94
CsMPK12	orange1.1g017231m	43.161	6.75
CsMPK13	orange1.1g042681m	39.285	5.80
CsMPK16	orange1.1g008639m	63.335	8.89
CsMPK17	orange1.1g044076m	68.574	7.50
CsMPK19	orange1.1g015019	48.058	8.80
CsMPK20	orange1.1g007177m	70.042	8.95
Coccomyxa subellipsoidea			

CsubMPK3	14112	39.761	9.10
CsubMPK4	11831	43.370	7.47
CsubMPK7	47764	44.105	8.20
CsubMPK15	54284	61.507	9.52
CsatMPK2	Cucsa.231940	44.646	6.91
CsatMPK3	Cucsa.213350	42.744	5.75
CsatMPK4-1	Cucsa.135720	42.929	6.80
CsatMPK4-2	Cucsa.303420	43.979	6.53
CsatMPK6	Cucsa.327650	46.313	5.84
CsatMPK7	Cucsa.106400	42.431	7.16
CsatMPK9-1	Cucsa.121850	54.919	8.37
CsatMPK9-2	Cucsa.111480	73.370	7.35
CsatMPK13	Cucsa.127090	42.563	5.22
CsatMPK16	Cucsa.363560	64.429	8.59
CsatMPK17	Cucsa.019360	53.806	7.45
CsatMPK19	Cucsa.273840	56.623	9.18
CsatMPK20-1	Cucsa.185440	68.386	9.08
CsatMPK20-2	Cucsa.139560	70.504	9.22
Eucalyptus grandis			
EgMPK1	Eucgr.F01598	42.687	6.84

EgMPK3	Eucgr.J00966	42.888	6.13
EgMPK4-1	Eucgr.E00103	43.226	6.29
EgMPK4-2	Eucgr.E00652	43.242	6.64
EgMPK6-1	Eucgr.G01599	45.416	5.76
EgMPK6-2	Eucgr.L00026	29.166	6.53
EgMPK9-1	Eucgr.B00871	70.064	7.23
EgMPK9-2	Eucgr.K02264	68.625	8.15
EgMPK13	Eucgr.J02112	43.259	5.21
EgMPK16	Eucgr.B01157	63.497	8.50
EgMPK17	Eucgr.B02905	57.792	7.70
EgMPK19	Eucgr.F01374	69.563	9.22
EgMPK20	Eucgr.A01974	70.162	8.57
Fragaria vesca			
FvMPK1	gene15192-v1.0-hybrid	42.644	6.53
FvMPK3	gene25390-v1.0-hybrid	47.445	6.67
FvMPK4-1	gene19238-v1.0-hybrid	42.672	6.53
FvMPK4-2	gene31827-v1.0-hybrid	46.667	6.87
FvMPK6	gene10128-v1.0-hybrid	44.713	6.04
FvMPK7	gene14943-v1.0-hybrid	42.641	8.10
FvMPK9	gene09401-v1.0-hybrid	78.449	7.81
FvMPK13	gene25407-v1.0-hybrid	70.005	6.41

FvMPK16	gene06108-v1.0-hybrid	63.072	8.31
FvMPK19	gene27365-v1.0-hybrid	65.337	9.08
FvMPK20	gene28706-v1.0-hybrid	97.433	9.17
Glycine max			
GmMPK1-1	Glyma06g03270	42.552	6.71
GmMPK1-2	Glyma04g03210	42.664	6.71
GmMPK3-1	Glyma12g07770	42.632	5.85
GmMPK3-2	Glyma11g15700	42.600	5.85
GmMPK4-1	Glyma11g02420	40.282	5.99
GmMPK4-2	Glyma08g02060	43.551	7.01
GmMPK4-3	Glyma05g37480	43.587	6.91
GmMPK4-4	Glyma01g43100	43.245	6.53
GmMPK4-5	Glyma09g39190	42.932	6.17
GmMPK4-6	Glyma07g07270	42.875	6.60
GmMPK4-7	Glyma16g03670	42.864	6.60
GmMPK4-8	Glyma18g47140	43.094	6.66
GmMPK6-1	Glyma02g15690	44.794	5.85
GmMPK6-2	Glyma07g32750	49.886	5.97
GmMPK7-1	Glyma08g12150	42.394	7.90
GmMPK7-2	Glyma05g28980	42.359	8.03
GmMPK9-1	Glyma08g05700	66.617	8.01

GmMPK9-2	Glyma05g33980	67.266	7.09
GmMPK9-3	Glyma09g30790	58.291	6.96
GmMPK9-4	Glyma07g11470	57.861	6.99
GmMPK13-1	Glyma12g07850	43.246	5.59
GmMPK13-2	Glyma11g15590	42.923	5.58
GmMPK16-1	Glyma17g02220	61.021	8.51
GmMPK16-2	Glyma13g28120	63.914	8.54
GmMPK16-3	Glyma15g10940	64.016	8.70
GmMPK19-1	Glyma13g33860	53.016	8.89
GmMPK19-2	Glyma15g38490	69.187	9.11
GmMPK20-1	Glyma14g03190	69.761	9.09
GmMPK20-2	Glyma02g45630	69.835	8.88
GmMPK20-3	Glyma08g42240	70.140	9.17
GmMPK20-4	Glyma18g12720	70.144	9.16
Gossipium raimondii			
GrMPK2-1	Gorai.009G199400	42.610	7.52
GrMPK2-2	Gorai.005G109500	42.721	6.84
GrMPK3-1	Gorai.009G104600	43.340	5.86
GrMPK3-2	Gorai.003G139900	43.025	5.93
GrMPK4-1	Gorai.004G159400	42.598	6.47
GrMPK4-2	Gorai.003G155700	42.741	6.36

GrMPK4-3	Gorai.008G120100	42.860	6.53
GrMPK4-4	Gorai.008G249800	43.336	7.05
GrMPK4-5	Gorai.001G158800	42.600	6.34
GrMPK4-6	Gorai.001G159000	43.071	6.68
GrMPK6-1	Gorai.005G123100	45.905	5.72
GrMPK6-2	Gorai.005G011100	45.506	5.93
GrMPK6-3	Gorai.011G132800	45.894	5.46
GrMPK7-1	Gorai.007G050000	42.460	7.52
GrMPK7-2	Gorai.003G012800	42.514	7.96
GrMPK7-3	Gorai.011G100600	42.349	7.88
GrMPK7-4	Gorai.008G065400	42.791	7.47
GrMPK9-1	Gorai.007G004400	67.704	6.44
GrMPK9-2	Gorai.008G289200	67.416	8.07
GrMPK13	Gorai.009G103800	42.696	5.28
GrMPK16	Gorai.005G035600	63.458	8.55
GrMPK17-1	Gorai.002G178700	57.926	7.23
GrMPK17-2	Gorai.005G161300	59.506	8.76
GrMPK19-1	Gorai.009G361300	68.215	9.12
GrMPK19-2	Gorai.007G332300	68.182	9.26
GrMPK19-3	Gorai.002G045300	67.491	9.10
GrMPK20-1	Gorai.012G041500	69.121	9.20

GrMPK20-2	Gorai.006G007700	71.502	8.85
Linum usitatissimum			
LuMPK2-1	Lus10010637	43.209	7.30
LuMPK2-2	Lus10033197	43.204	7.33
LuMPK3-1	Lus10036136	43.414	6.14
LuMPK3-2	Lus10018127	43.403	5.98
LuMPK3-3	Lus10038472	42.444	6.07
LuMPK3-4	Lus10023339	43.298	6.20
LuMPK4-1	Lus10007921	43.533	6.53
LuMPK4-2	Lus10024668	43.977	6.75
LuMPK4-3	Lus10017518	43.382	6.31
LuMPK4-4	Lus10032295	54.546	5.96
LuMPK4-5	Lus10036384	43.521	6.41
LuMPK4-6	Lus10028765	43.453	6.43
LuMPK6-1	Lus10027091	47.217	5.76
LuMPK6-2	Lus10008339	47.273	5.76
LuMPK7-1	Lus10014283	42.173	7.52
LuMPK7-2	Lus10025986	42.541	8.27
LuMPK9-1	Lus10027248	53.357	6.64
LuMPK9-2	Lus10038956	57.084	7.05
LuMPK16-1	Lus10041234	64.002	8.45

LuMPK16-2	Lus10021945	66.866	8.02
LuMPK17-1	Lus10021784	57.680	7.00
LuMPK17-2	Lus10034601	53.123	6.84
LuMPK18-1	Lus10005568	69.705	9.06
LuMPK18-2	Lus10013702	69.405	9.12
Malus domestica			
MdMPK1-1	MDP0000165532	42.321	6.53
MdMPK1-2	MDP0000210110	42.587	7.02
MdMPK1-3	MDP0000128473	42.321	6.53
MddPK3-1	MDP0000321850	42.661	5.98
MdMPK3-2	MDP0000237742	42.619	6.24
MdMPK3-3	MDP0000199036	42.661	5.98
MdMPK4-1	MDP0000766240	43.084	6.64
MdMPK4-2	MDP0000251955	42.976	6.60
MdMPK4-3	MDP0000321746	89.887	6.89
MdMPK4-4	MDP0000326020	41.551	6.47
MdMPK6-1	MDP0000340624	46.273	6.06
MdMPK6-2	MDP0000321308	45.807	6.27
MdMPK7-1	MDP0000807889	43.504	7.75
MdMPK7-2	MDP0000826016	43.502	7.94
MdMPK9	MDP0000294142	72.142	7.86

MdMdK13-1	MDP0000189383	57.028	6.04
MdMPK13-2	MDP0000173178	57.141	6.04
MdMPK13-3	MDP0000593502	42.790	5.22
MdMPK13-4	MDP0000422421	54.966	5.62
MdMPK16-1	MDP0000879089	66.606	8.03
MdMPK16-2	MDP0000170804	75.108	7.38
MdMPK17	MDP0000277562	66.371	6.98
MdMPK19-1	MDP0000169216	71.530	8.57
MdMPK19-2	MDP0000121116	68.920	8.99
MdMPK19-3	MDP0000195781	69.919	9.27
MdMPK19-4	MDP0000233021	71.700	9.07
MdMPK20-1	MDP0000188369	69.323	9.50
MdMPK20-2	MDP0000250639	98.915	9.08
Manihot esculenta			
MeMPK1	cassava4.1_009963m	42.641	7.49
MeMPK2	cassava4.1_009941m	42.627	7.16
MeMPK3	cassava4.1_010219m	42.122	5.56
MeMPK4-1	cassava4.1_009716m	43.352	7.38
MeMPK4-2	cassava4.1_009399m	44.593	6.84
MeMPK4-3	cassava4.1_010005m	42.638	6.80
MeMPK4-4	cassava4.1_009957m	42.959	6.80

MeMPK6	cassava4.1_008933m	46.048	5.65
MeMPK9-1	cassava4.1_004009m	67.359	8.58
MeMPK9-2	cassava4.1_003987m	67.631	6.87
MeMPK13	cassava4.1_009999m	42.677	5.05
MeMPK16-1	cassava4.1_004681m	64.048	8.54
MeMPK16-2	cassava4.1_004688m	63.977	8.78
MeMPK17-1	cassava4.1_006340m	55.956	7.27
MeMPK17-2	cassava4.1_006140m	57.015	7.32
MeMPK19	cassava4.1_005598m	58.713	9.07
MeMPK20	cassava4.1_004025m	68.515	9.12
Medicago truncatula			
MtMPK3	Medtr4g061130	41.096	5.84
MtMPK4-1	Medtr7g038040	42.854	6.25
MtMPK4-2	Medtr7g078690	42.966	7.08
MtMPK4-3	Medtr5g010030	43.248	6.53
MtMPK6	Medtr4g087620	44.394	5.76
MtMPK7-1	Medtr3g060350	32.097	8.93
MtMPK7-2	Medtr3g060390	38.461	6.98
MtMPK7-3	Medtr3g060330	42.578	8.21
MtMPK7-4	Medtr8g086000	48.403	6.88
MtMPK7-5	Medtr8g086010	49.022	8.32

MtMPK9	Medtr8g106960	70.184	7.29
MtMPK13	Medtr4g061320	43.034	5.12
MtMPK16	Medtr2g021330	64.268	8.44
MtMPK19	AC235677_37	68.634	9.37
MtMPK20-1	Medtr3g089510	69.934	9.17
MtMPK20-2	Medtr5g091680	57.664	8.85
MtMPK20-3	AC225528_53	69.299	9.20
Micromonas pusila			
MpMPK2	203785	42.681	5.91
MpMPK4	27380	46.920	5.00
MpMPK13	14294	44.050	7.30
MpMPK15	44271	40.960	7.30
Mimulus guttatus			
MgMPK1	mgv1a008410m	42.754	7.14
MgMPK4-1	mgv1a009790m	37.620	7.52
MgMPK4-2	mgv1a008501m	42.484	6.75
MgMPK9	mgv1a005228m	56.545	6.94
MgMPK17	mgv1a005103m	57.192	8.70
MgMPK20	mgv1a003659m	64.847	9.22
Oryza sativa			
OsMPK3	LOC_Os03g17700	42.995	5.78

OsMPK4-1	LOC_Os10g38950	42.772	6.41
OsMPK4-2	LOC_Os05g05160	44.584	6.89
OsMPK6	LOC_Os06g06090	44.857	5.75
OsMPK7	LOC_Os06g48590	42.244	7.30
OsMPK14	LOC_Os02g05480	42.469	7.16
OsMPK16-1	LOC_Os11g17080	56.966	8.23
OsMPK16-2	LOC_Os08g06060	61.762	8.62
OsMPK17-1	LOC_Os06g49430	65.944	7.45
OsMPK17-2	LOC_Os02g04230	58.281	8.15
OsMPK20-1	LOC_Os01g43910	69.400	8.93
OsMPK20-2	LOC_Os05g50560	38.616	9.32
OsMPK20-3	LOC_Os06g26340	64.523	8.57
OsMPK20-4	LOC_Os01g47530	67.682	9.21
OsMPK20-5	LOC_Os05g49140	67.008	8.99
OsMPK21-1	LOC_Os05g50120	66.487	6.93
OsMPK21-2	LOC_Os01g45620	57.099	7.43
Ostreococcus lucimarinus			
OlMPK6	C_Chr_9000126	45.634	5.79
OlMPK7	e_gwEuk.9.247	51.014	6.72
OlMPK9	gwEuk.8.109.1 gw	44.346	7.80
Panicum virgatum			

PvMPK3-1	Pavirv00035754m	42.882	5.65
PvMPK3-2	Pavirv00022561m	37.279	6.66
PvMPK4-1	Pavirv00033923m	42.569	6.41
PvMPK4-2	Pavirv00003084m	42.488	6.41
PvMPK4-3	Pavirv00026757m	44.135	6.92
PvMPK4-4	Pavirv00067074m	68.892	7.11
PvMPK6-1	Pavirv00041892m	44.822	5.66
PvMPK6-2	Pavirv00064097m	44.840	5.56
PvMPK7-1	Pavirv00007881m	42.323	7.11
PvMPK7-2	Pavirv00059104m	20.446	4.84
PvMPK14-1	Pavirv00021718m	42.468	7.01
PvMPK14-2	Pavirv00050004m	42.478	7.05
PvMPK16	Pavirv00037141m	62.054	8.63
PvMPK17-1	Pavirv00037180m	65.343	7.05
PvMPK17-2	Pavirv00047952m	52.968	7.04
PvMPK17-3	Pavirv00045770m	60.162	7.29
PvMPK17-4	Pavirv00062557m	65.144	6.87
PvMPK20-1	Pavirv00037472m	69.539	9.12
PvMPK20-2	Pavirv00069001m	70.749	9.29
PvMPK20-3	Pavirv00036824m	69.406	9.09
PvMPK20-4	Pavirv00038393m	67.298	9.25

PvMPK20-5	Pavirv00029083m	66.188	8.98
PvMPK20-6	Pavirv00062029m	67.123	9.06
PvMPK20-7	Pavirv00061344m	71.705	9.21
PvMPK21-1	Pavirv00045069m	58.477	8.18
PvMPK21-2	Pavirv00070777m	53.990	7.13
PvMPK21-3	Pavirv00054256m	57.494	7.26
Phaseolus vulgaris			
PvulMPK1	Phvul.009G061000	42.451	6.82
PvulMPK3	Phvul.011G071400	42.653	6.04
PvulMPK4-1	Phvul.010G102800	42.860	6.60
PvulMPK4-2	Phvul.002G292400	43.657	6.88
PvulMPK4-3	Phvul.002G159500	42.896	6.25
PvulMPK6	Phvul.003G059500	45.056	5.92
PvulMPK7	Phvul.002G237500	42.483	8.07
PvulMPK9	Phvul.004G151200	57.746	6.62
PvulMPK13	Phvul.011G070800	52.268	5.85
PvulMPK16-1	Phvul.003G095900	64.702	8.77
PvulMPK16-2	Phvul.006G155300	64.105	8.62
PvulMPK19	Phvul.005G069800	68.972	9.12
PvulMPK20-1	Phvul.008G191500	69.983	9.04
PvulMPK20-2	Phvul.006G033600	68.411	8.85

Physcomitrella patens			
PpMPK1	Pp1s207_63V6	42.362	6.88
PpMPK2	Pp1s138_117V6	42.488	6.88
PpMPK4-1	Pp1s99_26V6	73.735	5.72
PpMPK4-2	Pp1s59_325V6	43.487	5.68
PpMPK4-3	Pp1s149_39V6	42.831	5.92
PpMPK4-4	Pp1s29_285V6	78.019	6.00
PpMPK16-1	Pp1s80_71V6	60.843	8.83
PpMPK16-2	Pp1s87_157V6	61.028	8.84
Populus trichocarpa			
PtMPK1	Potri.002G032100	42.632	7.11
PtMPK2	Potri.005G231100	43.490	6.65
PtMPK3-1	Potri.009G066100	42.697	5.56
PtMPK3-2	Potri.001G271700	42.517	5.56
PtMPK4-1	Potri.002G162500	42.630	6.11
PtMPK4-2	Potri.014G088500	42.751	6.11
PtMPK6-1	Potri.017G010200	45.700	5.75
PtMPK6-2	Potri.007G139800	45.200	5.56
PtMPK7-1	Potri.007G020100	42.747	7.76
PtMPK7-2	Potri.005G119500	43.349	7.54
PtMPK9-1	Potri.015G040300	68.368	7.60

PtMPK9-2	Potri.012G048600	67.071	6.60
PtMPK12	Potri.003G131800	41.415	6.27
PtMPK16-1	Potri.010G029700	64.058	8.44
PtMPK16-2	Potri.008G200800	64.078	8.59
PtMPK17-1	Potri.010G112200	57.642	7.55
PtMPK17-2	Potri.008G130000	55.447	8.24
PtMPK19-1	Potri.011G102500	68.153	9.25
PtMPK19-2	Potri.001G381300	69.344	8.96
PtMPK20-1	Potri.002G059900	70.716	9.04
PtMPK20-2	Potri.005G201800	71.024	9.03
Prunus persica			
PperMPK1	ppa007332m		
PperMPK3	ppa007370m	42.610	5.98
PperMPK4-1	ppa007254m	43.188	6.68
PperMPK4-2	ppa007306m	42.824	6.53
PperMPK6	ppa006536m	46.410	6.18
PperMPK7	ppa007418m	42.406	8.21
PperMPK9-1	ppa002837m	71.140	7.15
PperMPK9-2	ppa003297m	66.379	7.06
PperMPK13	ppa007376m	42.489	5.42
PperMPK16	ppa005596m	51.114	8.58

PperMPK19	ppa003651m	63.745	9.42
PperMPK20	ppa002953m	70.595	9.00
Ricinus communis			
RcMPK2	30170.t000201	42.687	7.33
RcMPK3	29688.t000016	32.683	5.63
RcMPK4-1	30174.t000044	42.686	6.85
RcMPK4-2	30190.t000501	44.085	6.87
RcMPK6	29747.t000005	45.670	5.76
RcMPK7	29634.t000023	42.710	7.90
RcMPK9	28097.t000002	69.055	7.94
RcMPK13	28752.t000009	42.791	5.15
RcMPK15	29816.t000019	57.547	6.54
RcMPK16	29726.t000028	64.212	8.54
RcMPK19	29989.t000008	68.565	9.03
RcMPK20	29682.t000030	71.164	8.96
Selaginella moellendorffii			
SmMPK1	443152	43.290	6.84
SmMPK4	105143	43.064	5.32
SmMPK7	75282	42.534	6.41
SmMPK10	82767	43.905	8.96
SmMPK16-1	74687	54.865	9.24

SmMPK16-2	97841	47.581	8.77
Setaria italica			
SiMPK3	Si036218m	43.427	5.76
SiMPK4-1	Si036240m	42.294	6.41
SiMPK4-2	Si013899m	44.096	6.92
SiMPK6	Si006611m	44.470	5.75
SiMPK7	Si006708m	42.341	7.11
SiMPK14	Si017554m	42.412	7.01
SiMPK16-1	Si021645m	63.460	8.65
SiMPK16-2	Si026197m	61.170	8.62
SiMPK17-1	Si006144m	65.248	7.12
SiMPK17-2	Si016957m	55.553	7.60
SiMPK20-1	Si000725m	69.632	9.05
SiMPK20-2	Si021664m	61.957	9.40
SiMPK20-3	Si021560m	67.261	9.12
SiMPK20-4	Si000788m	67.374	9.29
SiMPK21-1	Si021565m	66.862	7.21
SiMPK21-2	Si004793m	54.716	8.10
Solanum lycopsersicum			
SlMPK1	Solyc04g080730	42.799	6.79
SlMPK3	Solyc06g005170	42.796	5.75

SlMPK4-1	Solyc05g049970	43.083	7.23
SlMPK4-2	Solyc01g094960	42.915	6.41
SlMPK5	Solyc08g081490	43.568	6.20
SlMPK6-1	Solyc08g014420	45.232	5.85
SlMPK6-2	Solyc12g019460	45.509	5.78
SlMPK7	Solyc02g084870	68.502	7.83
SlMPK9-1	Solyc06g068990	68.502	7.83
SlMPK9-2	Solyc12g040680	65.961	6.96
SlMPK13	Solyc11g072630	42.662	5.08
SlMPK15-1	Solyc04g007710	58.415	7.27
SlMPK15-2	Solyc05g008020	58.795	6.31
SlMPK16	Solyc01g080240	64.373	8.68
SlMPK19-1	Solyc10g007500	67.149	9.07
SlMPK19-2	Solyc07g062080	68.004	9.36
SlMPK20	Solyc07g056350	70.545	9.07
Solanum tuberosum			
StMPK1	PGSC0003DMG400003700	42.825	6.79
StMPK4-1	PGSC0003DMG400021649	43.056	7.23
StMPK4-2	PGSC0003DMG400012188	43.454	6.32
StMPK4-3	PGSC0003DMG401000057	42.817	6.29
StMPK7	PGSC0003DMG400003528	42.708	7.90

StMPK9	PGSC0003DMG402028796	69.042	7.49
StMPK15-1	PGSC0003DMG400005955	58.298	7.29
StMPK15-2	PGSC0003DMG400030492	58.650	6.46
StMPK16	PGSC0003DMG400004357	64.379	8.68
StMPK19-1	PGSC0003DMG400021253	67.184	9.13
StMPK19-2	PGSC0003DMG400007058	68.234	9.32
StMPK20	PGSC0003DMG400017345	69.709	9.16
Sorghum bicolor			
SbMPK3	Sb01g038750	43.411	5.85
SbMPK4-1	Sb01g030680	42.632	6.53
SbMPK4-2	Sb07g003810	44.044	6.73
SbMPK6	Sb10g003810	45.501	5.56
SbMPK7	Sb10g028780	42.395	7.30
SbMPK14	Sb04g003480	42.426	7.16
SbMPK16-1	Sb05g010000	47.510	7.98
SbMPK16-2	Sb09g003280	63.542	8.70
SbMPK17-1	Sb10g029400	65.511	7.21
SbMPK17-2	Sb04g002830	56.068	8.84
SbMPK20-1	Sb03g028740	69.947	9.07
SbMPK20-2	Sb09g029720	67.365	9.24
SbMPK20-3	Sb09g028690	67.215	9.13

SbMPK20-5	Sb03g030450	65.905	9.26
SbMPK21-1	Sb09g029370	66.829	7.18
SbMPK21-2	Sb03g029340	49.036	8.27
Thelluginella halophila			
ThMPK2	Thhalv10023530m	42.972	7.14
ThMPK3	Thhalv10002582m	42.478	6.09
ThMPK4	Thhalv10028738m	42.900	6.25
ThMPK5	Thhalv10028737m	43.215	5.59
ThMPK6	Thhalv10001439m	51.542	6.05
ThMPK7	Thhalv10025533m	41.869	7.42
ThMPK8	Thhalv10007165m	66.066	6.70
ThMPK9	Thhalv10020306m	71.218	7.03
ThMPK10	Thhalv10006460m	46.037	5.23
ThMPK12	Thhalv10001502m	42.577	7.95
ThMPK13	Thhalv10008001m	42.192	5.29
ThMPK16	Thhalv10013120m	64.915	8.79
ThMPK17	Thhalv10000832m	65.924	8.20
ThMPK18	Thhalv10011329m	68.485	8.97
ThMPK19	Thhalv10020339m	67.115	9.12
ThMPK20	Thhalv10016406m	68.156	9.21
Theobroma cacao			

TcMPK1	Thecc1EG034103	42.704	7.01
TcMPK3	Thecc1EG042004	43.002	5.93
TcMPK4-1	Thecc1EG005280	43.227	6.99
TcMPK4-2	Thecc1EG016328	42.828	6.47
TcMPK6	Thecc1EG019832	48.997	5.92
TcMPK7	Thecc1EG000305	38.621	8.05
TcMPK9	Thecc1EG012710	71.812	8.01
TcMPK13	Thecc1EG041990	42.928	5.23
TcMPK16	Thecc1EG020486	64.142	8.50
TcMPK17	Thecc1EG010874	56.854	8.62
TcMPK19	Thecc1EG032167	67.868	9.17
TcMPK20	Thecc1EG001868	70.916	9.14
Vitis venifera			
VvMPK1	GSVIVG01009766001	22.381	5.76
VvMPK3	GSVIVG01025105001	38.049	5.85
VvMPK4-1	GSVIVG01019406001	42.681	6.29
VvMPK4-2	GSVIVG01026984001	43.272	6.91
VvMPK6	GSVIVG01038192001	38.377	5.99
VvMPK7	GSVIVG01018883001	87.352	5.78
VvMPK9-1	GSVIVG01008408001	67.986	7.34
VvMPK9-2	GSVIVG01011749001	70.466	8.45

VvMPK13	GSVIVG01025091001	42.532	5.06
VvMPK16	GSVIVG01017873001	63.901	8.32
VvMPK19	GSVIVG01014081001	67.664	9.12
VvMPK20	GSVIVG01000784001	57.303	9.27
Volvox carteri			
VcMPK4-1	Vocar20007163m	45.142	6.18
VcMPK4-2	Vocar20000906m	43.526	7.49
VcMPK5	Vocar20007415m	48.358	8.85
VcMPK9	Vocar20004345m	52.499	8.50
VcMPK20	Vocar20011355m.g	71.410	7.77
Zea mays			
ZmMPK3-1	GRMZM2G053987	37.556	5.54
ZmMPK3-2	GRMZM2G017792	43.487	6.13
ZmMPK4-1	GRMZM2G127141	42.33	6.53
ZmMPK4-2	GRMZM2G123886	44.218	6.94
ZmMPK6-1	GRMZM2G002100	45.054	5.55
ZmMPK6-2	GRMZM2G020216	44.933	5.65
ZmMPK7	GRMZM2G048455	42.207	7.52
ZmMPK14	GRMZM2G062914	42.465	7.01
ZmMPK16	GRMZM2G089484	63.576	8.60
ZmMPK17-1	GRMZM2G306028	57.226	7.05

ZmMPK17-2	GRMZM2G374088	55.737	8.43
ZmMPK17-3	GRMZM2G135904	33.829	7.54
ZmMPK20-1	GRMZM2G131334	55.944	8.79
ZmMPK20-2	GRMZM2G163861	67.622	9.29
ZmMPK20-4	GRMZM2G122335	67.012	9.29
ZmMPK20-5	GRMZM2G007848	67.150	9.38
ZmMPK20-6	GRMZM2G034052	65.394	6.71
ZmMPK21-1	GRMZM2G062761	68.135	7.09
ZmMPK21-2	GRMZM2G375975	55.857	7.09
