# Supplementary material for: Identification of new members of the MAPK gene family in plants shows diverse conserved domains and novel activation loop variants
Source: BMC Genomics. 2015 Feb 6;16(1):58. doi: 10.1186/s12864-015-1244-7 (PMC4363184; doi:10.1186/s12864-015-1244-7)
Supplement: Additional file 3: — Additional data file showing the average amino acid composition of plant MAPKs. [file 12864_2015_1244_MOESM3_ESM.pdf]

Additional data file 3.

Additional data file showing average amino acid composition of plant MAPKs.

Amino Acids	Average amino acid composition of CBL gene
Ala	6.91
Cys	1.66
Asp	6.01
Glu	6.73
Phe	4.45
Gly	4.86
His	3.29
Ile	6.24
Lys	5.87
Leu	9.63
Met	2.35
Asn	4.30
Pro	6.25
Gln	3.64
Arg	6.63
Ser	6.12
Thr	4.65
Val	5.70
Trp	0.70
Tyr	3.91
