# Supplementary figures and images for: Identification of new members of the MAPK gene family in plants shows diverse conserved domains and novel activation loop variants
Source: BMC Genomics. 2015 Feb 6;16(1):58. doi: 10.1186/s12864-015-1244-7 (PMC4363184; doi:10.1186/s12864-015-1244-7)

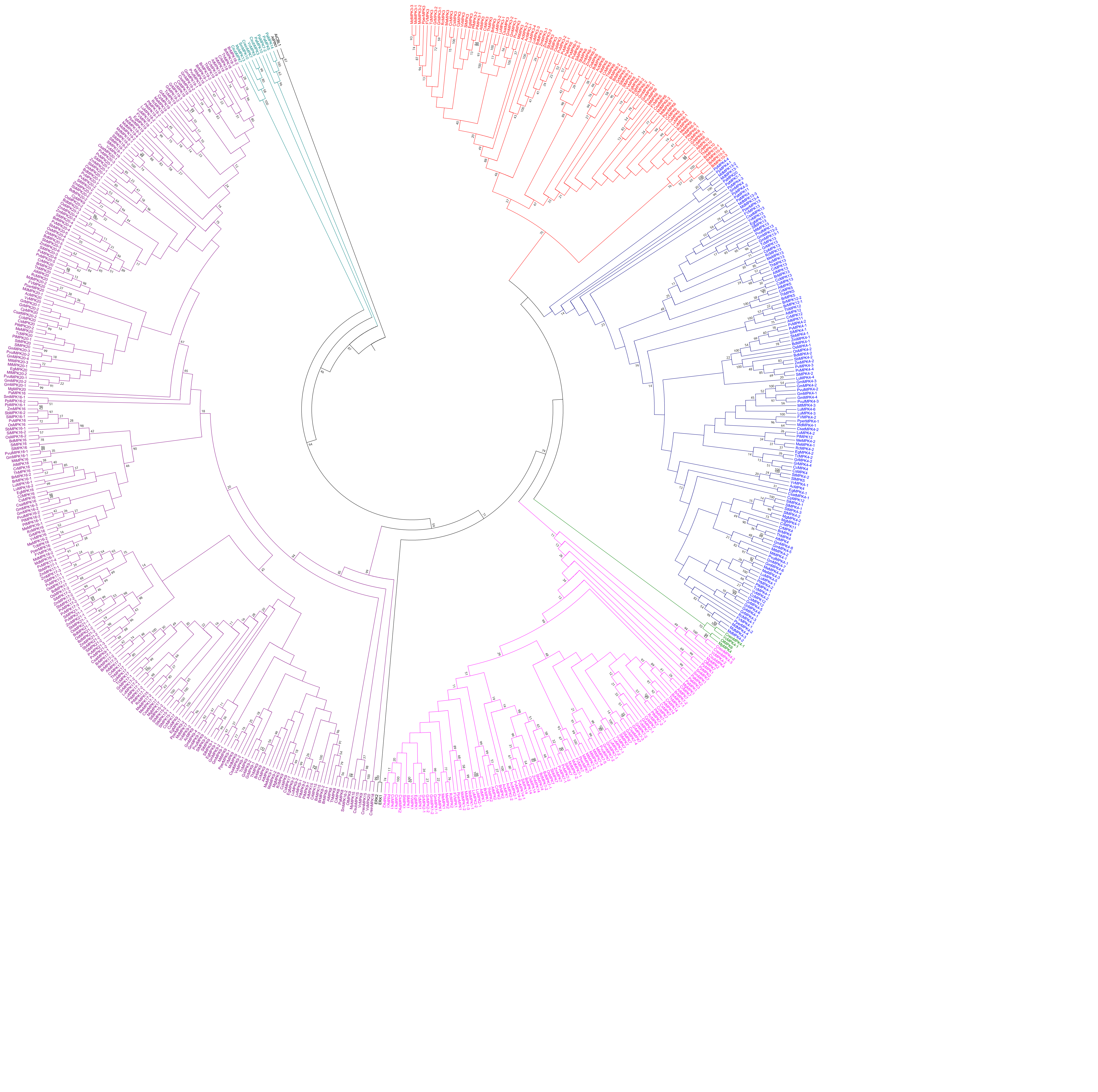

Supplement: Additional file 5: — Additional data file showing the phylogenetic tree of plant MAPKs. Red color indicates group A , blue color group B, pink color group C, purple color group D, teal color group E and green color represent group F MAPKs. [file 12864_2015_1244_MOESM5_ESM.pdf]
